# Synthesis and bioevaluation of *N*,4-diaryl-1,3-thiazole-2-amines as tubulin inhibitors with potent antiproliferative activity

**DOI:** 10.1371/journal.pone.0174006

**Published:** 2017-03-23

**Authors:** Maolin Sun, Qile Xu, Jingwen Xu, Yue Wu, Yueting Wang, Daiying Zuo, Qi Guan, Kai Bao, Jian Wang, Yingliang Wu, Weige Zhang

**Affiliations:** 1 Key Laboratory of Structure-Based Drug Design and Discovery, Ministry of Education, Shenyang Pharmaceutical University, Shenyang, China; 2 Department of Pharmacology, Shenyang Pharmaceutical University, Shenyang, China; 3 Gordon Center for Medical Imaging, Division of Nuclear Medicine and Molecular Imaging, Department of Radiology, Massachusetts General Hospital and Harvard Medical School, Boston, Massachusetts, United States of America; Johns Hopkins University School of Medicine, UNITED STATES

## Abstract

A series of *N*,4-diaryl-1,3-thiazole-2-amines containing three aromatic rings with an amino linker were designed and synthesized as tubulin inhibitors and evaluated for their antiproliferative activity in three human cancer cell lines. Most of the target compounds displayed moderate antiproliferative activity, and *N*-(2,4-dimethoxyphenyl)-4-(4-methoxyphenyl)-1,3-thiazol-2-amine (**10s**) was determined to be the most potent compound. Tubulin polymerization and immunostaining experiments revealed that **10s** potently inhibited tubulin polymerization and disrupted tubulin microtubule dynamics in a manner similar to CA-4. Moreover, **10s** effectively induced SGC-7901 cell cycle arrest at the G_2_/M phase in both concentration- and time-dependent manners. The molecular docking results revealed that **10s** could bind to the colchicine binding site of tubulin.

## Introduction

**Fig 1:**
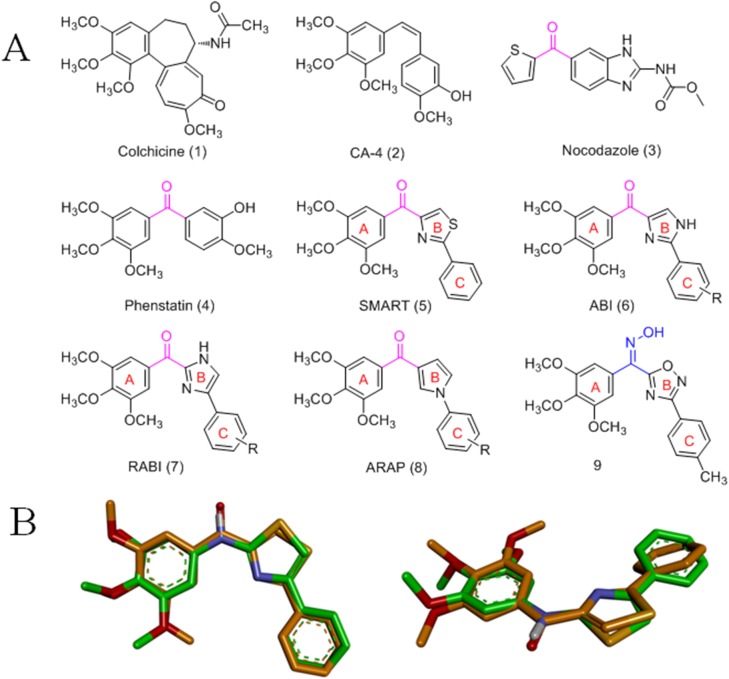
**(A) Structures of known tubulin inhibitors. (B) Superposition of the skeletal structure of target compounds 10a**-**m and SMART.** All density functional theory calculations and geometry optimizations were performed using the Gaussian 09 software.

Microtubules are key structural components in eukaryotic cells and play a crucial role in a number of cellular functions, including regulation of motility, cell division, organelle transport, maintenance of cell morphology and signal transduction [1,2]. Tubulin-binding drugs disrupt microtubule/tubulin dynamics by binding to distinct sites such as taxol, vinca alkaloid and colchicine binding sites and arrest cells during mitosis, leading to cell death [3–5]. In contrast to macromolecular paclitaxel and vinblastine, most novel colchicine binding site inhibitors (CBSIs) have high potency, relatively simple and diverse chemical structures for optimization, and selective toxicity toward tumor vasculature [6]. Given the success of paclitaxel and vinblastine, research efforts have been focused on developing CBSIs for cancer treatment, and a number of effective CBSIs are currently being investigated in clinical studies [7]. Some representative CBSIs include the natural products colchicine (**1**), combretastatin A-4 (CA-4, **2**) and nocodazole (**3**), shown in Fig 1A [8–12]. Phenstatin (**4**) is a CA-4 analog in which the double bond of CA-4 is replaced with a carbonyl linker [13]. The common structural features of CBSIs include the 3,4,5-Trimethoxyphenyl unit and the appropriate distance and certain dihedral angle between the aromatic rings. These were proposed as important structural features that are responsible for their antiproliferative and antitubulin activities [6].

The CBSIs 4-substituted methoxybenzoyl-aryl-thiazole (SMART, **5**), 2-aryl-4-benzoyl-imidazole (ABI, **6**), 4-aryl-2-benzoyl-imidazoles (RABI, **7**) and 3-aroyl-1-arylpyrrole (ARAP, **8**) are the representative synthetic CBSIs that exhibit potent antiproliferative activity (Fig 1A) [14–17]. Furthermore, a set of oxadiazole CBSIs with an oxime linker between the A-ring and the B-ring was previously reported by our group (**9**, Fig 1A) [18]. The structures of these CBSIs feature three aromatic rings (A-, B- and C- rings) and an sp^2^ hybridized linker between the A-ring and B-ring (Fig 1A).

2-Aminothiazoles and derivatives are seen in many bioactive scaffolds and exhibit a variety of biological properties, including anticancer activity [19]. Furthermore, CBSIs containing an sp^3^ amino linker have also been reported [20]. In our continued research into novel CBSIs [18, 21, 22], we hypothesized that the sp^2^ linker between the A- and B-rings could be replaced by a sp^3^ amino linker and that the substitutions on the A- and C-rings could be different groups. As expected from calculations using the Gaussian 09 software, the skeletal structure of target compounds superimposed well with that of SMART (Fig 1B). In this study, we report the verification of our hypothesis through the synthesis and biological evaluation of the designed target CBSIs (Table 1). In addition, the preliminary structure-activity relationships and the mechanism of the representative compound **10s** are investigated and determined.

**Table 1:** Molecular structures of compounds 10a-v.

Structures	R_1_	R_2_	R_3_	R_4_	R_5_	R_6_	R_7_	R_8_	R_9_
**10a**	H	OCH_3_	OCH_3_	OCH_3_	H	H	CH_3_	H	H
**10b**	H	OCH_3_	OCH_3_	OCH_3_	H	H	OCH_3_	H	H
**10c**	H	OCH_3_	OCH_3_	OCH_3_	H	H	F	H	H
**10d**	H	OCH_3_	OCH_3_	OCH_3_	H	H	Cl	H	H
**10e**	H	OCH_3_	OCH_3_	OCH_3_	H	H	Br	H	H
**10f**	H	OCH_3_	OCH_3_	OCH_3_	H	H	NO_2_	H	H
**10g**	H	OCH_3_	OCH_3_	OCH_3_	H	H	OCH_3_	OCH_3_	H
**10h**	H	OCH_3_	OCH_3_	OCH_3_	H	H	OCH_3_	F	H
**10i**	H	OCH_3_	OCH_3_	OCH_3_	H	H	OCH_3_	NO_2_	H
**10j**	H	OCH_3_	OCH_3_	OCH_3_	H	H	OCH_3_	NH_2_	H
**10k**	H	OCH_3_	OCH_3_	OCH_3_	H	H	OCH_3_	OBn	H
**10l**	H	OCH_3_	OCH_3_	OCH_3_	H	H	OCH_3_	OH	H
**10m**	H	OCH_3_	OCH_3_	OCH_3_	H	H	F	F	H
**10n**	H	OCH_3_	H	OCH_3_	H	H	OCH_3_	H	H
**10o**	H	H	OCH_3_	H	H	OCH_3_	OCH_3_	OCH_3_	H
**10p**	H	CH_3_	CH_3_	H	H	H	OCH_3_	NO_2_	H
**10q**	H	CH_3_	CH_3_	H	H	H	OCH_3_	NH_2_	H
**10r**	OCH_3_	H	OCH_3_	H	H	H	CH_3_	H	H
**10s**	OCH_3_	H	OCH_3_	H	H	H	OCH_3_	H	H
**10t**	OCH_3_	H	OCH_3_	H	H	H	OCH_3_	H	OCH_3_
**10u**	OCH_3_	H	OCH_3_	H	CH_3_	H	OCH_3_	H	H
**10v**	OCH_3_	H	OCH_3_	H	Ac	H	OCH_3_	H	H

## Results and discussion

### Chemistry

**Fig 2:**
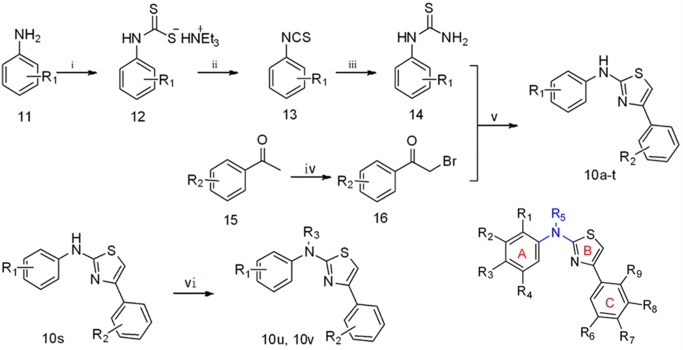
**Reagents and conditions.** (i) CS_2_, Et_3_N, Et_2_O, 25°C; (ii) I_2_, Et_3_N, EtOAc, 0°C; (iii) NH_3_·H_2_O; (iv) CuBr_2_, CHCl_3_, EtOAc; (v) EtOH, MW 80°C; (vi) 1) CH_3_I, DMF, 25°C or 2) Ac_2_O, 50°C.

The target compounds contained a 2-aminothiazole (2-AT) scaffold that has been widely used by medicinal chemists in drug discovery research. Few drugs containing a 2-AT core (e.g., mirabegron, riluzole, cefotaxime and meloxicam) have been launched in the market [23]. In our designed compounds, the amino group played a vital role in maintaining the spatial configuration and biological activity of target compounds. Target compounds were prepared as outlined in Fig 2. In brief, the condensation of commercially aniline derivatives **11** with carbon disulfide in the presence of triethylamine gave the corresponding dithiocarbamatets **12** at room temperature at an 80–96% yield [24]. Subsequently, dithiocarbamates **12** were used for the synthesis of aryl thioamides **14** using an efficient one-pot method [25]. More speculatively, dithiocarbamates **12** in ethyl acetate were incubated with triethylamine at 0°C. Iodine was then added pinch-wise over a period of 15 minutes to yield phenylisothiocyanates **13**, which were then reacted with ammonium hydroxide to obtain the key intermediates aryl thioureas **14** at room temperature. Alternatively, the commercially available starting acetophenones **15** were subjected to α-bromination with copper dibromide in refluxing chloroform-ethyl acetate to produce α-bromoacetophenones **16** at 75–95% yield. Finally, α-bromoacetophenones **16** were reacted with aryl thioureas **14** to generate the desired compounds **10a-t** in ethanol within 5 min under microwave irradiation (150 W, 80°C) in the absence of catalysts [26]. The compound **10u** was obtained by a simple reaction of the compound **10s** with methyl iodide in anhydrous DMF, and the compound **10v** was obtained by an acetylation reaction of compound **10s** with acetic anhydride.

### Biological studies

#### In vitro antiproliferative activity

Gastric cancer and lung cancers are the two most common cancers and the two most frequent causes of cancer-related deaths [27]. HT-1080 is a fibrosarcoma cell line which has been used extensively in biomedical research [28]. So we chose these three human cancer cell lines (gastric adenocarcinoma SGC-7901 cells, lung adenocarcinoma A549 cells and fibrosarcoma HT-1080 cells) for antiproliferative activity. To evaluate the ability of various 2-aminothiazole derivatives to inhibit cancer cell growth, the target compounds **10a-v**, reference compounds CA-4 (**2**), nocodazole (**3**) and SMART (**5**) were screened for antiproliferative activity in three human cancer cell lines using a standard MTT assay (Table 2). The reported IC_50_ values are the average of at least three independent experiments. As illustrated in Table 2, most of the target compounds showed moderate anti-proliferative activities with potencies in the μM range. Comparison of the IC_50_ values of the corresponding compounds **10a-m** with 3,4,5-trimethoxy (R_2_, R_3_, R_4_ = OMe) substitution revealed that only the 3-hydroxy-4-methoxy-substituted compound **10l** significantly inhibited growth of the HT-1080 cell line at sub-micromolar concentrations. The target compounds contain halide substituents that has been widely used by medicinal chemists in CBSI research, so we synthesis these compounds to evaluate the potential antiproliferative activities. Unexpectedly, we found that these compounds (**10c**, **10d**, **10e**, **10h**, **10m**) showed only moderate antiproliferative activities. Interestingly, the most significant enhancement of antiproliferative activity was observed in the amino-linked compounds where the A ring accommodates the 2,4-dimethoxy substitutions. *N*-(2,4-dimethoxyphenyl)-4-(4-methoxyphenyl)-1,3-thiazol-2-amine **10s** exhibited the most potent antiproliferative activity, with IC_50_ values between 0.36 and 0.86 μM in the three cancer cell lines. However, the introduction of a methyl (**10u**) or acetyl (**10v**) group at the N position of the 2-aminothiazole skeleton resulted in reduced activity compared to the corresponding compound (**10s**).

**Table 2:** Antiproliferative activity of compounds 10a-v, CA-4 (2), Nocodazole (3) and SMART (5).

	Antiproliferative activity (IC_50_± SD, μM) ^a^		
Compounds	SGC-7901	A549	HT-1080
**10a**	1.12 ± 0.13	1.32 ± 0.12	1.17 ± 0.19
**10b**	7.72 ± 0.26	26.8 ± 2.2	26.8 ± 1.2
**10c**	27.7 ± 4.9	16.1 ± 1.1	7.40 ± 0.42
**10d**	8.22 ± 0.17	1.80 ± 0.19	1.26 ± 0.25
**10e**	2.30 ± 0.32	3.18 ± 0.16	6.61 ± 0.18
**10f**	>100	23.6 ± 2.3	>100
**10g**	4.89 ± 0.15	5.36 ± 0.16	6.94 ± 0.14
**10h**	4.92 ± 0.12	6.17 ± 0.32	5.48 ± 0.21
**10i**	9.11 ± 0.62	9.25 ± 0.33	6.24 ± 0.24
**10j**	1.17 ± 0.17	6.54 ± 0.16	4.69 ± 0.62
**10k**	13.0 ± 2.1	13.1 ± 1.1	5.01 ± 0.18
**10l**	1.36 ± 0.09	3.21 ± 0.17	0.77 ± 0.11
**10m**	10.1 ± 1.1	26.4 ± 1.3	8.46 ± 0.17
**10n**	0.82 ± 0.06	9.62 ± 0.17	3.36 ± 0.11
**10o**	19.5 ± 2.4	17.7 ± 1.6	12.7 ± 1.9
**10p**	9.89 ± 0.18	12.8 ± 1.3	25.8 ± 3.5
**10q**	14.8 ± 1.5	10.9 ± 3.2	4.89 ± 0.25
**10r**	1.6 ± 2.2	1.82 ± 0.12	1.95 ± 0.13
**10s**	**0.36 ± 0.19**	**0.86± 0.07**	**0.58 ± 0.13**
**10t**	10.1 ± 1.0	11.4 ± 1.3	2.46 ± 0.17
**10u**	28.2 ± 1.9	19.0 ± 2.0	9.39 ± 0.21
**10v**	23.2 ± 0.11	35.7 ± 0.08	56.6 ± 1.0
CA-4 (**2**)^b^	0.025 ± 0.012	0.019 ± 0.011	0.032 ± 0.006
Nocodazole (**3**)^b^	0.24 ± 0.07	0.15 ± 0.09	0.12 ± 0.08
SMART (**5**)^b^	0.045 ± 0.013	0.059 ± 0.012	0.032 ± 0.009

^a^ IC_50_: Concentration of the compound (μM) that resulted in 50% cell growth inhibition after 72 h of drug exposure, as determined by the MTT assay. Each experiment was carried out in triplicate.

^b^ Used as a positive control.

#### Tubulin polymerization

**Fig 3:**
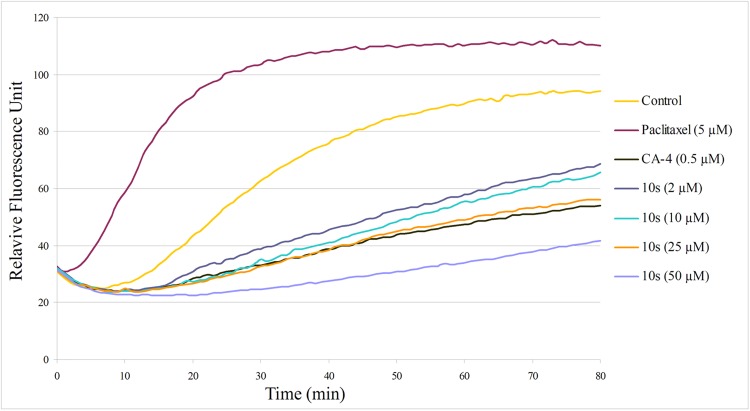
Effects of 10s on tubulin polymerization. Tubulin was pre-incubated for 1 min with **10s** at 2 μM, 10 μM, 25 μM and 50 μM, CA-4 at 0.5 μM, paclitaxel at 5 μM or vehicle DMSO (control) at room temperature before GTP was added to start the tubulin polymerization reactions. The reaction was monitored at 37°C.

CBSIs exert their biological activity by inhibiting tubulin assembly. To confirm that the antiproliferative activities of these compounds were related to the microtubule system, the most active compound **10s** was evaluated for tubulin polymerization inhibition compared to the positive control CA-4 and the negative control paclitaxel. As shown in Fig 3, both CA-4 and **10s** inhibited tubulin assembly, and compound **10s** inhibited tubulin polymerization in a dose-dependent manner. However, compound **10s** displayed less active antitubulin activity than did CA-4 (IC_50_ = 26.8 μM and 0.64 μM, respectively). In contrast, paclitaxel enhanced the rate of tubulin polymerization compared with untreated cells. These results suggested that compound **10s** interfered with microtubule polymerization.

#### Analysis of immunofluorescence staining

**Fig 4:**
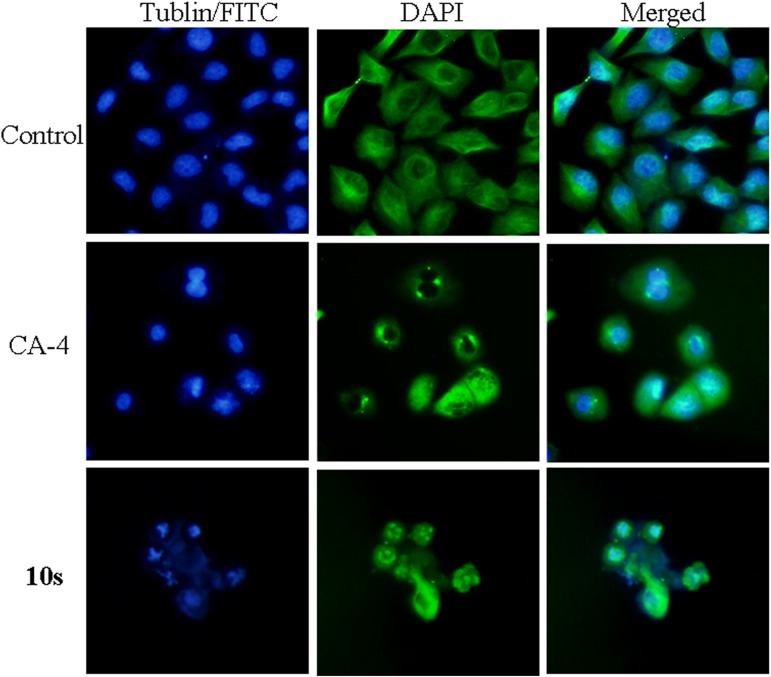
Immunostaining of tubulin assembly in SGC-7901 cells. SGC-7901 cells were treated with **10s** (0.72 μM) for 48 h. Tubulin assembly was stained with FITC (left panel) and DAPI (middle panel). A merge of the two panels is shown on the right. Images were taken using a confocal microscope.

Colchicine binding site inhibitors could inhibit tubulin assembly and suppress microtubule formation. With the goal of ascertaining the mechanism of action of compound **10s**, we decided to use a 0.72 μM drug concentration for the cell microtubule organization studies. As shown in Fig 4, the fibrous microtubules (green) and the cell nucleus (blue) were visualized upon the immunofluorescence staining of SGC-7901 cells. These results implied that **10s** and CA-4 induced significant defects in tubulin assembly in the treated cells, leading to the formation of abnormal mitotic spindles in comparison with the control. These results further confirmed that tubulin was the molecular target for compound **10s**.

#### Cell cycle study

**Fig 5:**
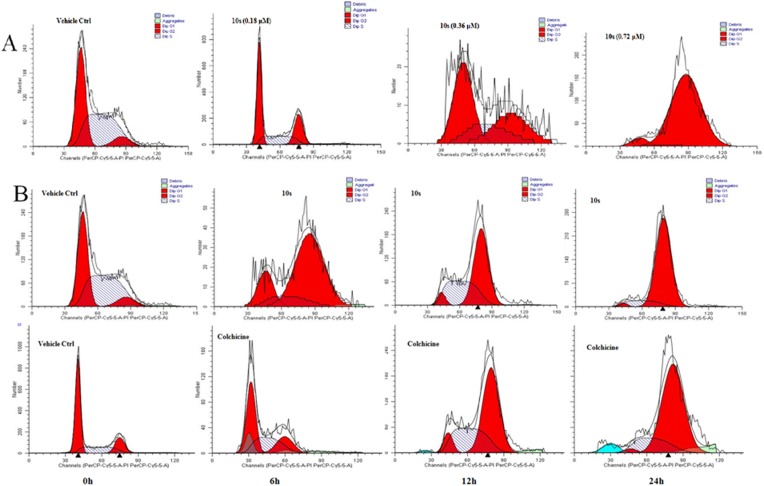
(A) **10s** caused G2/M phase arrest in a concentration-dependent manner in SGC-7901 cells. Cells were treated with **10s** (0.18, 0.36, 0.72 μM) for 24 h. (B) **10s** and CA-4 induced G2/M phase arrest in a time-dependent manner. SGC-7901 cells were treated with **10s** (0.72 μM) or CA-4 (0.05 μM) for 6, 12 and 24 h.

Because tubulin inhibitors can arrest dividing cells in the G2/M phase of the cell cycle, the effect of **10s** on the cell cycle progression of SGC-7901 cells was investigated by flow cytometry analysis. As demonstrated in Fig 5A, **10s** caused a dramatic increase in G2/M phase cell counts in a concentration-dependent manner. Furthermore, treatment of SGC-7901 cells with **10s** (0.72 μM) or CA-4 (0.05 μM) from 6 to 24 h induced a dramatic increase in the number of cells in the G2/M phase in a time-dependent manner. These results further demonstrated that **10s** was a tubulin inhibitor.

#### Molecular modeling

**Fig 6:**
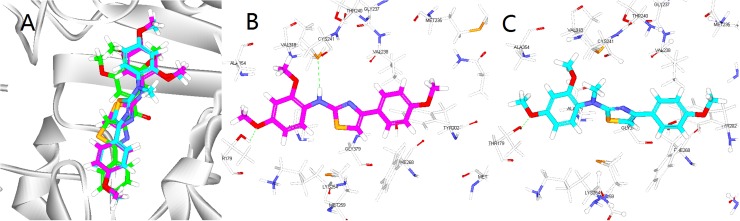
(A). The binding mode of compound **10s** (violet), **10u** (cyan) and SMART (green) in the colchicine binding site of tubulin. (B).Overlay of **10s** in the binding site. Hydrogen bonds are shown using green dashed lines and the distance is less than 3 Å. (C).Overlay of **10u** in the binding site.

Molecular modeling studies were performed to investigate the potential binding ability of the most active compound **10s** and a moderate compound **10u** to the colchicine binding site of α- and β-tubulin. The molecular modeling study was performed using the CDOCKER protocol in Discovery Studio 3.0 with the default settings to predict the binding mode of our ligand (PDB: 3HKD). These studies showed that **10s**, **10u** and SMART can occupy the colchicine binding site of tubulin (Fig 6). The trimethoxyphenyl moiety of SMART and the dimethoxyphenyl moiety of **10s** and **10u** in the A-rings extended toward the α- and β-tubulin interfaces. Several amino acids of β-tubulin formed hydrophobic interactions with compounds **10s** and **10u**. The thiol group of Cys β241 formed a hydrogen bond with the nitrogen atom of the amino linker and the methoxy oxygen atom in ring A of **10s** formed a hydrogen bond with the hydroxyl group of Thr α179. However, the thiol group of Cys β241 could not form a hydrogen bond with the nitrogen atom of **10u** due to the methyl group on nitrogen atom. This may be the reason that the activities of **10u** was less active than **10s**. The docking study results revealed that **10s** could bind the colchicine binding site of tubulin.

#### QSAR model

**Fig 7:**
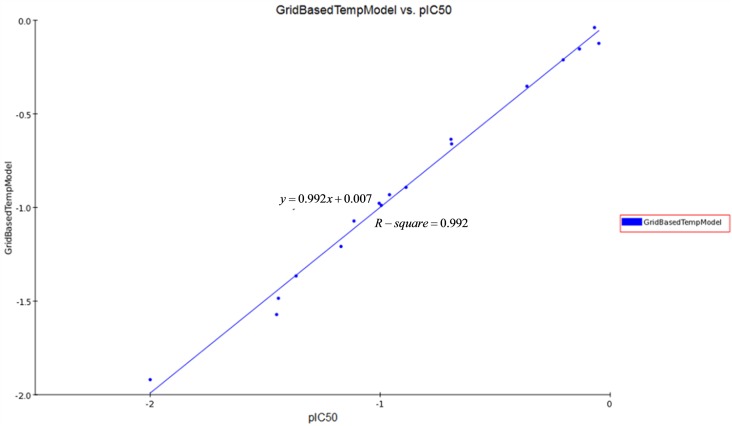
We used a linear fitting curve to compare the predicted pIC50 values (modeled based on a known dataset of 21 defined compounds in SGC-7901 cells) with the experimental pIC50 values. *y* = 0.992*x* + 0.007, *R* – *square* = 0.992.

**Fig 8:**
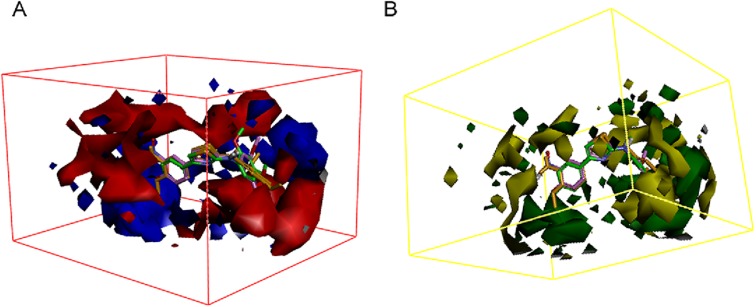
(A). 3D-QSAR model coefficients on electrostatic potential grids. Blue represents positive coefficients; red represents negative coefficients. (B). 3D-QSAR model coefficients on Van der Waals grids. Green represents positive coefficients; yellow represents negative coefficients. (For interpretation of the references to color in this figure legend, the reader is referred to the web version of this article.)

By using the Create 3D QSAR protocol in Discovery Studio 3.0, twenty-one synthesized compounds with definite IC_50_ values against SGC-7901 were selected as the model dataset. By convention, we used the pIC_50_ scale (−log IC_50_), in which higher values indicate exponentially greater potency, to measure inhibitory activity. The training set and testing set were chosen by the Diverse Molecules method in Discovery Studio 3.0. To ensure a good alignment, we chose the alignment conformation of each molecule with the lowest energy in the docked results of the CDOCKER protocol. We applied the alignment of the substructure **10a** before building the QSAR model. The correlation coefficient r^2^ between the observed activity of the testing set and the training set was found to be 0.992; therefore, the QSAR model that we built was acceptable (greater than 0.5, Fig 7). Additionally, the molecules that aligned with the iso-surfaces of the 3D-QSAR model (Organic & Biomolecular Chemistry Page 10 of 349 coefficients) on Van der Waals grids (Fig 8A) and electrostatic potential grids (Fig 8B) were listed. Our electrostatic map indicated red contours around regions where high electron density (negative charge) was expected to increase activity, and blue contours represented areas where low electron density (partial positive charge) was expected to increase activity. Similarly, our steric map indicated areas where steric bulk was predicted to increase (green) or decrease (yellow) activity. It was widely acceptable that a better inhibitor based on the 3D QSAR model should have stronger Van der Waals attractions in the green areas and a polar group in the blue electrostatic potential areas (which were dominant close to the skeleton). Thus, this promising model would provide a guideline to design and optimize more effective tubulin inhibitors based on the N,4-diaryl-1,3-thiazole-2-amines skeleton and pave the way for us to further study these compounds in the future.

## Conclusions

In summary, we designed and synthesized a series of *N*,4-diaryl-1,3-thiazole-2-amines containing three aromatic rings with an amino linker as novel CBSIs. Most of the target compounds showed moderate anti-proliferative activity in different cancer cells lines with potencies in the μM range. Among them, *N*-(2,4-dimethoxyphenyl)-4-(4-methoxyphenyl)-1,3-thiazol-2-amine, **10s**, was found to be the most potent compound with IC_50_ values at the submicromolar level in three human cancer cell lines. Tubulin polymerization and immunostaining experiments revealed that **10s** potently inhibited tubulin polymerization and disrupted tubulin microtubule dynamics in a manner similar to CA-4. Moreover, **10s** effectively induced SGC-7901 cell cycle arrest at the G_2_/M phase in both concentration- and time-dependent manners. Molecular docking results revealed that **10s** could bind the colchicine binding site of tubulin. Taken together, the present study indicates that the utilization of an sp^3^ amino linker to replace the sp^2^ carbonyl linker can be one of the future directions of novel CBSI synthesis and is deserving of further study. Investigation of the modification of these novel CBSIs and mechanistic studies for anticancer properties are underway in our lab. We will continue to optimize and refine the modification of these compounds and will then select one or two active compounds for in vivo testing.

## Experimental section

### Chemistry

All solvents and chemical materials were obtained from commercially available sources and were used without purification. The microwave reactions were performed on a discover-sp single mode microwave reactor from CEM Corporation. The progress of reactions was monitored by TLC using silica gel plates under UV light. NMR spectra were recorded on a Bruker AVANCE 400, or 600 spectrometer (^1^H, 400 MHz, 600 MHz; ^13^C, 100 MHz, 150 MHz), in CDCl_3_ or DMSO-d_6_ (TMS was used as an internal standard). Chemical shifts are expressed as parts per million downfield from tetramethylsilane. Mass spectra (MS) were measured on an Agilent 1100-sl mass spectrometer with an electrospray ionization source. Melting points were measured on a hot stage microscope (X-4, Beijing Taike Ltd.) and are uncorrected.

#### General synthetic procedures for *N*,4-diaryl-1,3-thiazole-2-amines 10a-v

The α-bromoketones **16** (1.0 mmol) and aryl thioamides **14** (1.0 mmol) were dissolved in ethanol (5 mL). The mixture was heated to 80°C for 5 min under microwave irradiation (discover-sp, CEM Corporation, 150 W). After the reaction was completed, the mixture was poured into water and extracted with methylene chloride. The organic layer was washed with deionized water, dried over anhydrous sodium sulfate, and filtered, and the solvent was then evaporated under reduced pressure to yield compounds **10a-t**. The compound **10u** was obtained by a simple reaction of the compound **10s** with methyl iodide in anhydrous DMF, and the compound **10v** was obtained by an acetylation reaction of compound **10s** with acetic anhydride.

*N-(3*,*4*,*5-trimethoxyphenyl)-4-(4-methyphenyl)-1*,*3-thiazol-2-amine (****10a****)*. Yellow solid; yield: 81%; M.p.: 179–181°C; ^1^H-NMR (600 MHz, CDCl_3_): δ 7.75 (d, *J* = 7.9 Hz, 2H), 7.19 (d, *J* = 7.9 Hz, 2H), 6.76 (s, 1H), 6.59 (s, 2H), 3.80 (s, 3H), 3.71 (s, 6H), 2.36 (s, 3H); ^13^C-NMR (150 MHz, CDCl_3_): δ 165.6, 153.6 (2C), 151.0, 137.8, 136.7, 133.6, 131.7, 129.3 (2C), 126.0 (2C), 100.5 (2C), 96.2, 60.9, 55.8 (2C), 21.1; ESI-MS: m/z = 357.2 [M+H]^+^, 379.1 [M+Na]^+^.

*N-(3*,*4*,*5-trimethoxyphenyl)-4-(4-methoxyphenyl)-1*,*3-thiazol-2-amine (****10b****)*. Brown solid; yield: 79%; M.p.: 65–67°C; ^1^H-NMR (600 MHz, CDCl_3_): δ 7.77 (d, *J* = 8.7 Hz, 2H), 6.90 (d, *J* = 8.7 Hz, 2H), 6.66 (s, 1H), 6.62 (s, 2H), 3.82 (s, 3H), 3.81 (s, 3H), 3.77 (s, 6H); ^13^C-NMR (150 MHz, CDCl_3_): δ 165.4, 159.4, 153.6 (2C), 150.6, 136.6, 133.7, 127.3 (2C), 127.2, 114.0 (2C), 99.5, 96.3 (2C), 60.9, 55.9 (2C), 55.2; ESI-MS: m/z = 373.2 [M+H]^+^, 395.1 [M+Na]^+^.

*N-(3*,*4*,*5-trimethoxyphenyl)-4-(4-fluorophenyl)-1*,*3-thiazol-2-amine (****10c****)*. Pale brown solid; yield: 70%; M.p.: 134–136°C; ^1^H-NMR (600 MHz, CDCl_3_): δ 7.80 (m, 2H), 7.06 (m, 2H), 6.73 (s, 1H), 6.64 (s, 2H), 3.82 (s, 3H), 3.81 (s, 6H); ^13^C-NMR (150 MHz, CDCl_3_): δ 165.5, 162.5 (d, *J* = 248.0 Hz), 153.7 (2C), 150.0, 136.4, 133.9, 130.6 (d, *J* = 3.0 Hz), 127.7 (d, *J* = 8.0 Hz, 2C), 115.5 (d, *J* = 21.7 Hz, 2C), 101.0, 96.5 (2C), 61.0, 56.0 (2C); ESI-MS: m/z = 361.2 [M+H]^+^, 383.1 [M+Na]^+^, 399.1 [M+K]^+^.

*N-(3*,*4*,*5-trimethoxyphenyl)-4-(4-chlorophenyl)-1*,*3-thiazol-2-amine (****10d****)*. Pale yellow solid; yield: 77%; M.p.: 149–151°C; ^1^H-NMR (600 MHz, CDCl_3_): δ 7.77 (d, *J* = 8.5 Hz, 2H), 7.34 (d, *J* = 8.5 Hz, 2H), 6.79 (s, 1H), 6.61 (s, 2H), 3.82 (s, 3H), 3.77 (s, 6H); ^13^C-NMR (150 MHz, CDCl_3_): δ 165.7, 153.7 (2C), 149.9, 136.4, 133.9, 133.6, 132.9, 128.8 (2C), 127.2 (2C), 101.8, 96.5 (2C), 61.0, 55.9 (2C); ESI-MS: m/z = 377.1 [M+H]^+^, 399.1 [M+Na]^+^.

*N-(3*,*4*,*5-trimethoxyphenyl)-4-(4-bromophenyl)-1*,*3-thiazol-2-amine (****10e****)*. Yellow solid; yield: 82%; M.p.: 144–146°C; ^1^H-NMR (600 MHz, CDCl_3_): δ 7.69 (d, *J* = 8.4 Hz, 2H), 7.51 (d, *J* = 8.4 Hz, 2H), 6.79 (s, 1H), 6.65 (s, 2H), 3.83 (s, 9H); ^13^C-NMR (150 MHz, CDCl_3_): δ 165.6, 153.7 (2C), 149.9, 136.3, 134.0, 133.2, 131.7 (2C), 127.5 (2C), 121.8, 101.9, 96.6 (2C), 61.0, 56.0 (2C); ESI-MS: m/z = 421.0 [M+H]^+^.

*N-(3*,*4*,*5-trimethoxyphenyl)-4-(4-nitrophenyl)-1*,*3-thiazol-2-amine (****10f****)*. Orange solid; yield: 73%; M.p.: 98–99°C; ^1^H-NMR (600 MHz, CDCl_3_): δ 8.32 (d, *J* = 8.0 Hz, 2H), 7.57 (d, *J* = 8.0 Hz, 2H), 6.36 (s, 2H), 5.99 (s, 1H), 3.84 (s, 9H); ^13^C-NMR (150 MHz, CDCl_3_): δ 160.1, 153.8 (2C), 147.9, 138.4, 137.5, 133.9, 129.1 (2C), 126.4, 124.1 (2C), 98.7, 98.3 (2C), 60.9, 55.9 (2C); ESI-MS: m/z = 388.2 [M+H]^+^, 410.2 [M+Na]^+^.

*N-(3*,*4*,*5-trimethoxyphenyl)-4-(3*,*4-dimethoxyphenyl)-1*,*3-thiazol-2-amine (****10g****)*. Brown solid; yield: 75%; M.p.: 54–56°C; ^1^H-NMR (600 MHz, CDCl_3_): δ 7.38 (m, 2H), 6.88 (d, *J* = 8.7 Hz, 1H), 6.67 (s, 1H), 6.66 (s, 2H), 3.92 (s, 3H), 3.90 (s, 3H), 3.82 (s, 9H); ^13^C-NMR (150 MHz, CDCl_3_): δ 165.4, 153.7 (2C), 150.1, 149.1, 148.9, 136.2, 134.0, 127.0, 118.5, 111.2, 109.3, 99.7, 96.5 (2C), 60.9, 56.0 (2C), 55.9, 55.8; ESI-MS: m/z = 403.2 [M+H]^+^, 425.1 [M+Na]^+^.

*N-(3*,*4*,*5-trimethoxyphenyl)-4-(3-fluoro-4-methoxyphenyl)-1*,*3-thiazol-2-amine (****10h****)*. Brown red solid; yield: 76%; M.p.: 148–150°C; ^1^H-NMR (600 MHz, CDCl_3_): δ 7.56 (s, 1H), 7.54 (s, 1H), 6.93–6.96 (m, 1H), 6.67 (s, 1H), 6.65 (s, 2H), 3.90 (s, 3H), 3.82 (s, 3H), 3.80 (s, 6H); ^13^C-NMR (150 MHz, CDCl_3_): δ 165.5, 153.6 (2C), 152.3 (d, *J* = 245.0 Hz), 149.6, 147.4 (d, *J* = 10.8 Hz), 136.4, 133.8, 127.9 (d, *J* = 6.9 Hz), 121.8 (d, *J* = 2.2 Hz), 113.8 (d, *J* = 19.7 Hz), 113.3, 100.6, 96.5 (2C), 60.9, 56.2, 55.9 (2C); ESI-MS: m/z = 391.2 [M+H]^+^, 413.2 [M+Na]^+^, 429.1 [M+K]^+^.

*N-(3,4,5-trimethoxyphenyl)-4-(3-nitro-4-methoxyphenyl)-1,3-thiazol-2-amine (****10i****)*. Yellow solid; yield: 84%; M.p.: 173-174°C; ^1^H-NMR (600 MHz, CDCl_3_): δ 8.35 (d, *J* = 1.5 Hz, 1H), 7.94 (dd, *J* = 8.7 Hz, *J* = 1.5Hz, 2H), 7.08 (d, *J* = 8.7 Hz, 1H), 6.76 (s, 1H), 6.75 (s, 2H), 3.97 (s, 3H), 3.86 (s, 6H), 3.83 (s, 3H); ^13^C-NMR (150 MHz, CDCl_3_): δ 165.2, 153.7 (2C), 152.4, 148.5, 139.4, 136.3, 133.9, 131.2, 127.4, 123.4, 113.6, 101.6, 96.3 (2C), 61.0, 56.6, 56.0 (2C); ESI-MS: m/z = 418.2 [M+H]^+^.

*N-(3*,*4*,*5-trimethoxyphenyl)-4-(3-amino-4-methoxyphenyl)-1*,*3-thiazol-2-amine (****10j****)*. Yellow solid; yield: 65%; M.p.: 166–167°C; ^1^H-NMR (600 MHz, CDCl_3_): δ 7.20 (m, 2H), 6.79 (d, *J* = 8.1 Hz, 1H), 6.62 (s, 2H), 6.61 (s, 1H), 3.87 (s, 3H), 3.81 (s, 3H), 3.79 (s, 6H); ^13^C-NMR (150 MHz, CDCl_3_): δ 165.4, 153.6 (2C), 151.0, 147.3, 136.6, 136.1, 133.7, 127.5, 116.5, 112.7, 110.3, 99.5, 96.4 (2C), 60.9, 55.9 (2C), 55.5; ESI-MS: m/z = 388.2 [M+H]^+^, 410.2 [M+Na]^+^.

*N-(3*,*4*,*5-trimethoxyphenyl)-4-(3-benzyloxy-4-methoxyphenyl)-1*,*3-thiazol-2-amine (****10k****)*. Red solid; yield: 74%; M.p.: 77–78°C; ^1^H-NMR (600 MHz, DMSO-d_6_): δ 10.20 (s, 1H), 7.61 (d, *J* = 2.0 Hz, 1H), 7.51 (dd, *J* = 8.3 Hz, *J* = 2.0 Hz, 1H), 7.49 (d, *J* = 7.3 Hz, 2H), 7.41 (m, 2H), 7.35 (d, *J* = 7.3 Hz, 1H), 7.19 (s, 1H), 7.16 (s, 2H), 7.03 (d, *J* = 8.3Hz, 1H), 5.12 (s, 2H), 3.82 (s, 6H), 3.80 (s, 3H), 3.63 (s, 3H); ^13^C-NMR (150 MHz, DMSO-d_6_): δ 163.3, 153.4 (2C), 150.3, 149.3, 148.2, 137.9, 137.4, 132.2, 128.8 (2C), 128.3 (2C), 128.0, 119.0, 112.6, 111.6, 111.6, 101.3, 95.0 (2C), 70.4, 60.5, 56.0, 56.0 (2C); ESI-MS: m/z = 479.2 [M+H]^+^, 501.1 [M+Na]^+^, 517.1 [M+K]^+^.

*N-(3*,*4*,*5-trimethoxyphenyl)-4-(3-hydroxy-4-methoxyphenyl)-1*,*3-thiazol-2-amine (****10l****)*. Red solid; yield: 73%; M.p.: 98–100°C; ^1^H-NMR (600 MHz, CDCl_3_): δ 7.37 (d, *J* = 1.9 Hz, 1H), 7.36 (dd, *J* = 8.3 Hz, *J* = 1.9 Hz, 2H), 6.85 (d, *J* = 8.3 Hz, 1H), 6.65 (s, 2H), 6.64 (s, 1H), 3.90 (s, 3H), 3.82 (s, 6H), 3.82 (s, 3H); ^13^C-NMR (150 MHz, CDCl_3_): δ 165.3, 153.6 (2C), 150.5, 146.6, 145.6, 136.6, 133.7, 128.0, 118.1, 112.3, 110.7, 100.0, 96.4 (2C), 60.9, 56.0 (2C), 55.9; ESI-MS: m/z = 389.1 [M+H]^+^, 777.3 [2M+H]^+^, 799.3 [2M+Na]^+^.

*N-(3,4,5-trimethoxyphenyl)-4-(3,4-difluorophenyl)-1,3-thiazol-2-amine (****10m****)*. Yellow solid; yield: 64%; M.p.: 153-155°C; ^1^H-NMR (600 MHz, CDCl_3_): δ 7.70-7.71 (m, 1H), 7.62-7.65 (m, 1H), 7.14-7.18 (m, 1H), 6.74 (s, 1H), 6.70 (s, 2H), 3.86 (s, 6H), 3.83 (s, 3H); ^13^C-NMR (150 MHz, CDCl_3_): δ 165.3, 153.7 (2C), 149.0, 136.3, 134.0, 132.2, 130.9, 128.8, 121.8, 117.4 (d, *J* = 16.8 Hz), 115.0 (d, *J* = 18.9 Hz), 102.0, 96.5 (2C), 61.0, 56.0 (2C); ESI-MS: m/z = 379.1 [M+H]^+^.

*N-(3*,*5-dimethoxyphenyl)-4-(4-methoxyphenyl)-1*,*3-thiazol-2-amine (****10n****)*. Pale yellow solid; yield: 63%; M.p.: 150–151°C; ^1^H-NMR (600 MHz, CDCl_3_): δ 7.79 (d, *J* = 8.7 Hz, 2H), 6.91 (d, *J* = 8.7 Hz, 2H), 6.71 (s, 1H), 6.51 (d, *J* = 2.1 Hz, 1H), 6.15 (m, 1H), 3.82 (s, 3H), 3.70 (s, 6H); ^13^C-NMR (150 MHz, CDCl_3_): δ 164.5, 161.4 (2C), 159.4, 150.6, 142.1, 130.2, 127.3 (2C), 114.0 (2C), 100.1, 96.2 (2C), 95.1, 55.2, 55.2 (2C); ESI-MS: m/z = 343.2 [M+H]^+^, 365.2 [M+Na]^+^.

*N-(4-methoxyphenyl)-4-(3*,*4*,*5-trimethoxyphenyl)-1*,*3-thiazol-2-amine (****10o****)*. Khaki solid; yield: 66%; M.p.: 66–68°C; ^1^H-NMR (600 MHz, CDCl_3_): δ 7.20 (d, *J* = 8.8 Hz, 2H), 7.02 (s, 2H), 6.82 (d, *J* = 8.8 Hz, 2H), 6.63 (s, 1H), 3.84 (s, 6H), 3.83 (s, 3H), 3.77 (s, 3H); ^13^C-NMR (150 MHz, CDCl_3_): δ 167.4, 156.3, 153.2 (2C), 137.9, 133.6, 130.3, 121.9 (2C), 114.7, 114.5 (2C), 103.4 (2C), 100.5, 60.8, 56.0 (2C), 55.4; ESI-MS: m/z = 373.2 [M+H]^+^, 395.1 [M+Na]^+^.

*N-(3*,*4-dimethphenyl)-4-(3-nitro-4-methoxyphenyl)-1*,*3-thiazol-2-amine (****10p****)*. Red solid; yield: 63%; M.p.: 166–167°C; ^1^H-NMR (600 MHz, CDCl_3_): δ 8.27 (s, 1H), 7.94 (d, *J* = 8.7 Hz, 1H), 7.08 (s, 2H), 7.01 (m, 2H), 6.70 (s, 1H), 3.93 (s, 3H), 2.22 (s, 3H), 2.20 (s, 3H); ^13^C-NMR (150 MHz, CDCl_3_): δ 166.3, 152.2, 148.4, 139.4, 137.8, 137.8, 132.1, 131.6, 130.4, 127.5, 123.2, 120.7, 116.5, 113.5, 101.4, 56.5, 19.9, 19.0; ESI-MS: m/z = 356.1 [M+H]^+^, 378.1 [M+Na]^+^.

*N-(3*,*4-dimethphenyl)-4-(3-amino-4-methoxyphenyl)-1*,*3-thiazol-2-amine (****10q****)*. Brown solid; yield: 68%; M.p.: 146–148°C; ^1^H-NMR (600 MHz, CDCl_3_): δ 7.18 (s, 1H), 7.16 (m, 1H), 7.09 (m, 2H), 7.06 (s, 1H), 6.76 (d, *J* = 8.8 Hz, 1H), 6.55 (s, 1H), 3.85 (s, 3H), 2.23 (s, 3H), 2.22 (s, 3H); ^13^C-NMR (150 MHz, CDCl_3_): δ 165.8, 150.4, 147.3, 138.0, 137.7, 136.1, 131.7, 130.3, 127.3, 120.5, 116.5, 116.3, 112.9, 110.3, 99.2, 55.5, 19.9, 19.0; ESI-MS: m/z = 326.1 [M+H]^+^, 348.1 [M+Na]^+^, 364.0 [M+K]^+^.

*N-(2*,*4-dimethoxyphenyl)-4-(4-methphenyl)-1*,*3-thiazol-2-amine (****10r****)*. Brown solid; yield: 75%; M.p.: 65–66°C; ^1^H-NMR (600 MHz, CDCl_3_): δ 7.91 (d, *J* = 8.4 Hz, 1H), 7.75 (d, *J* = 8.0 Hz, 2H), 7.21 (d, *J* = 8.0 Hz, 2H), 6.71 (s, 1H), 6.54 (m, 2H), 3.87 (s, 3H), 3.82 (s, 3H) 2.38 (s, 3H); ^13^C-NMR (150 MHz, CDCl_3_): δ 165.1, 155.8, 151.1, 149.6, 137.5, 131.8, 129.2 (2C), 125.9 (2C), 123.7, 118.6, 103.7, 100.4, 99.1, 55.7, 55.5, 21.2; ESI-MS: m/z = 327.2 [M+H]^+^, 349.1 [M+Na]^+^.

*N-(2*,*4-dimethoxyphenyl)-4-(4-methoxyphenyl)-1*,*3-thiazol-2-amine (****10s****)*. Brown red solid; yield: 80%; M.p.: 98–99°C; ^1^H-NMR (600 MHz, CDCl_3_): δ 7.92 (d, *J* = 8.4 Hz, 1H), 7.79 (d, *J* = 8.7 Hz, 2H), 6.93 (d, *J* = 8.7 Hz, 2H), 6.62 (s, 1H), 6.53 (m, 2H), 3.85 (s, 3H), 3.83 (s, 3H), 3.81 (s, 3H); ^13^C-NMR (150 MHz, CDCl_3_): δ 165.0, 159.2, 155.7, 151.0, 149.5, 127.7, 127.3 (2C), 123.8, 118.5, 113.8 (2C), 103.7, 99.4, 99.1, 55.6, 55.5, 55.2; ESI-MS: m/z = 343.2 [M+H]^+^, 365.1 [M+Na]^+^, 381.1 [M+K]^+^.

*N-(2*,*4-dimethoxyphenyl)-4-(2*,*4-dimethoxyphenyl)-1*,*3-thiazol-2-amine (****10t****)*. Brown solid; yield: 65%; M.p.: 151–153°C; ^1^H-NMR (600 MHz, CDCl_3_): δ 8.13 (d, *J* = 8.4 Hz, 1H), 7.95 (d, *J* = 8.5 Hz, 1H), 7.13 (s, 1H), 6.59 (dd, *J* = 8.5 Hz, *J* = 1.9 Hz, 1H), 6.53 (m, 3H), 3.91 (s, 3H), 3.86 (s, 3H), 3.84 (s, 3H), 3.81 (s, 3H); ^13^C-NMR (150 MHz, CDCl_3_): δ 163.1, 160.0, 157.9, 155.5, 149.3, 146.8, 130.7, 124.0, 118.2, 116.8, 104.5, 104.0, 103.7, 99.0, 98.6, 55.6, 55.5, 55.3, 55.3; ESI-MS: m/z = 373.2 [M+H]^+^, 395.1 [M+Na]^+^.

*N-(2*,*4-dimethoxyphenyl)-4-(4-methoxyphenyl)-N-methyl-1*,*3-thiazol-2-amine (****10u****)*. NaH (5.4 mmol) was added to a stirred solution of **10s** (2.8 mmol) in DMF (5 mL), and the mixture was stirred at 25°C for 2 min. MeI (2.8 mmol) was added dropwise, and the mixture was stirred at 25°C for 1 h. The reaction was quenched with saturated aqueous NH_4_Cl solution (15 mL), and the mixture was extracted with EtOAc (3 × 15 mL). The combined organic fraction was dried and the solvent was evaporated. The crude solid was purified by column chromatography to yield **10u**. White solid; yield: 53%; M.p.: 126–128°C; ^1^H-NMR (600 MHz, CDCl_3_): δ 7.81 (d, *J* = 8.8 Hz, 2H), 7.27 (s, 1H), 6.92 (d, *J* = 8.8 Hz, 2H), 6.58 (d, *J* = 2.6 Hz, 1H), 6.52 (dd, *J* = 8.6 Hz, *J* = 2.6 Hz, 1H), 6.48 (s, 1H), 3.85 (s, 3H), 3.84 (s, 3H) 3.80 (s, 3H), 1.27 (s, 3H); ^13^C-NMR (150 MHz, CDCl_3_): δ 171.6, 160.4, 158.9, 156.9, 151.2, 130.1, 128.4, 127.7, 127.2 (2C), 113.7 (2C), 104.7, 100.2, 99.7, 55.6, 55.5, 55.2, 39.3; ESI-MS: m/z = 357.2 [M+H]^+^, 379.1 [M+Na]^+^, 395.1 [M+K]^+^.

*N-(2*,*4-dimethoxyphenyl)-4-(4-methoxyphenyl)-N-acetyl-1*,*3-thiazol-2-amine (****10v****)*. A flask immersed in a room temperature oil bath was charged with **10s** (2 mmol) and Ac_2_O (10 mL) and heated to 50°C. After completion of the reaction, the reaction was quenched upon addition of 50 g of ice. A yellow precipitate formed, which was filtered and washed with H_2_O. The crude solid was purified by column chromatography to give pure **10v**. Yellow solid; yield: 54%; M.p.: 146–148°C; ^1^H-NMR (600 MHz, CDCl_3_): δ 7.58 (d, *J* = 8.8 Hz, 2H), 7.22 (d, *J* = 9.2 Hz, 1H), 7.03 (s, 1H), 6.82 (d, *J* = 8.8 Hz, 2H), 6.60 (m, 2H), 3.89 (s, 3H), 3.77 (s, 3H), 3.74 (s, 3H), 2.06 (s, 3H); ^13^C-NMR (150 MHz, CDCl_3_): δ 171.0, 161.2, 159.1, 155.9, 148.8, 130.7, 127.8, 127.1 (2C), 122.2, 113.7 (2C), 106.5, 104.8, 99.6, 55.7, 55.5, 55.2, 22.9; ESI-MS: m/z = 385.2 [M+H]^+^, 407.1 [M+Na]^+^.

### Biology

MTT assay: The SGC-7901, A549 and HT-1080 cell lines were purchased from the American Type Culture Collection (ATCC, Manassas, VA, USA). The in vitro antiproliferative activities of CA-4 (**2**), Nocodazole (**3**) and all of the target compounds were determined with an MTT assay following a previously reported method [21,22].

Tubulin assembly assay: The effect of **10s** on the polymerization of purified brain tubulin was determined using a fluorescence-based tubulin polymerization assay kit (Cat. #BK011P, Cytoskeleton, Inc., USA) following a previously reported method [22].

Immunofluorescence assay: Immunostaining was performed to detect the microtubule-associated tubulin protein after exposure to CA-4 (**2**) or the investigated compound **10s** following a previously reported method [21,22].

Cell cycle analysis: SGC-7901 cells (8 × 10^4^ cells) were incubated with various concentrations of CA-4 (**2**), **10s** or 0.05% DMSO for the indicated times. The cells were collected by centrifugation, washed with PBS and fixed in ice-cold 70% ethanol. The fixed cells were harvested by centrifugation and resuspended in 500 μl of PBS containing 1 mg/mL RNase. After 30 min of incubation at 37°C, the cells were stained with 50 mg/mL propidium iodide (PI) at 4°C in the dark for 30 min. The samples were then analyzed by FACScan flow cytometry (Becton-Dickinson, Franklin Lakes, NJ, USA). The experiments were repeated at least three times.

Molecular modeling: The molecular modeling studies were performed using Accelrys Discovery Studio 3.0. The crystal structure of tubulin complexed with DAMA-colchicine (PDB: 3HKD) was retrieved from the RCSB Protein Data Bank (http://www.rcsb.org/pdb). In the docking process, the protein protocol was prepared via several operations, including the standardization of atom names, insertion of missing atoms in residues and removal of alternate conformations, insertion of missing loop regions based on SEQRES data, optimization of short and medium sized loop regions with the Looper Algorithm, minimization of remaining loop regions, calculation of pK, and protonation of the structure. The receptor model was then typed with the CHARMm force field, and a binding sphere with a radius of 9.0 Å was defined with the original ligand (DAMA-colchicine) as the binding site. The **10s** was drawn with Chemdraw and fully minimized using the CHARMm force field. Finally, **10s** was docked into the binding site using the CDOCKER protocol with the default settings.

QSAR Model: In the model, 80% of the target compounds were utilized as a training set for QSAR modeling. The remaining 20% were chosen as a test subset using the same protocol. The literature-based inhibitory activity values of the known compounds [IC_50_ of SGC-7901 cells] were initially converted into the minus logarithmic scale [pIC_50_] and then used for subsequent QSAR analysis as the response variable. In Discovery Studio, the CHARMm force field was applied and the electrostatic potential together with the Van der Waals potential were treated as separate terms. As the electrostatic potential probe, A+ le point change was used while distance-dependent dielectric constant was used to mimic the solvent effect. A carbon atom with a radius of 1.73 Å was used as a probe for the Van der Waals potential. A Partial Least-Squares (PLS) model was built using energy grids as descriptors. QSAR models were built by using the Create 3D QSAR Model protocol in Discovery Studio 3.0.

## Supporting information

S1 File **(1). Synthesis.** 1. General synthetic procedures for aryl thioamides 14. 2. General synthetic procedures for α-bromoacetophenones 16. **(2). Contents:** 1H-NMR and 13C-NMR spectra of all target compounds.
(PDF)

## References

[1] Stephens RE, Edds KT. Microtubules: structure, chemistry, and function. Physiol Rev. 1976; 56(4): 709–777. 790425 10.1152/physrev.1976.56.4.709

[2] Etienne-Manneville S. From signaling pathways to microtubule dynamics: the key players. Curr Opin Cell Biol. 2010; 22(1): 104–111. 10.1016/j.ceb.2009.11.008 20031384

[3] Kavallaris M. Microtubules and resistance to tubulin-binding agents. Nat Rev Cancer. 2010; 10(3): 194–204. 10.1038/nrc2803 20147901

[4] Dumontet C, Jordan MA. Microtubule-binding agents: a dynamic field of cancer therapeutics. Nat Rev Drug Discov. 2010; 9(10): 790–803. 10.1038/nrd3253 20885410 PMC3194401

[5] Jordan MA, Wilson L. Microtubules as a target for anticancer drugs. Nat Rev Cancer. 2004; 4(4): 253–265. 10.1038/nrc1317 15057285

[6] Dong M, Liu F, Zhou H, Zhai S, Yan B. Novel Natural Product- and Privileged Scaffold-Based Tubulin Inhibitors Targeting the Colchicine Binding Site. Molecules. 2016; 21(10): 1375–1401. 10.3390/molecules21101375 PMC6273505 27754459

[7] Carlson RO. New tubulin targeting agents currently in clinical development. Expert Opin. Expert Opin Investig Drugs. 2008; 17(5): 707–722. 10.1517/13543784.17.5.707 18447597

[8] Graening T, Schmalz HG. Total syntheses of colchicine in comparison: a journey through 50 years of synthetic organic chemistry. Angew Chem Int Ed Engl. 2004; 43(25): 3230–3256. 10.1002/anie.200300615 15213947

[9] Pettit GR, Singh SB, Niven ML, Hamel E, Schmidt JM. Isolation, structure, and synthesis of combretastatins A-1 and B-1, potent new inhibitors of microtubule assembly, derived from Combretum caffrum. J Nat Prod. 1987; 50(1): 119–131. 3598594 10.1021/np50049a016

[10] Pettit GR, Singh SB, Hamel E, Lin CM, Alberts DS, Garcia-Kendall D. Isolation and structure of the strong cell growth and tubulin inhibitor combretastatin A-4. Experientia. 1989; 45(2): 209–211. 2920809 10.1007/BF01954881

[11] Chaudhary A, Pandeya SN, Kumar P, Sharma PP, Gupta S, Soni N, et al Combretastatin a-4 analogs as anticancer agents. Mini Rev Med Chem. 2007; 7(12): 1186–1205. 18220974 10.2174/138955707782795647

[12] Attia SM. Molecular cytogenetic evaluation of the mechanism of genotoxic potential of amsacrine and nocodazole in mouse bone marrow cells. J Appl Toxicol. 2013; 33(6): 426–433. 10.1002/jat.1753 22081495

[13] Pettit GR, Toki B, Herald DL, Verdier-Pinard P, Boyd MR, Hamel E, et al Antineoplastic agents. 379. Synthesis of phenstatin phosphate. J Med Chem. 1998; 41(10): 1688–1695. 10.1021/jm970644q 9572894

[14] Lu Y, Li CM, Wang Z, Ross CR, Chen J, Dalton JT, et al Discovery of 4-substituted methoxybenzoyl-aryl-thiazole as novel anticancer agents: synthesis, biological evaluation, and structure-activity relationships. J Med Chem. 2009; 52(6): 1701–1711. 10.1021/jm801449a 19243174 PMC2760094

[15] Chen J, Wang Z, Li CM, Lu Y, Vaddady PK, Meibohm B, et al Discovery of novel 2-aryl-4-benzoyl-imidazoles targeting the colchicines binding site in tubulin as potential anticancer agents. J Med Chem. 2010; 53(20): 7414–7427. 10.1021/jm100884b 20919720 PMC2964152

[16] Xiao M, Ahn S, Wang J, Chen J, Miller DD, Dalton JT, et al Discovery of 4-Aryl-2-benzoyl-imidazoles as tubulin polymerization inhibitor with potent antiproliferative properties. J Med Chem. 2013; 56(8): 3318–3329. 10.1021/jm4001117 23547728 PMC3668676

[17] La Regina G, Bai R, Coluccia A, Famiglini V, Pelliccia S, Passacantilli S, Mazzoccoli C, et al New Pyrrole Derivatives with Potent Tubulin Polymerization Inhibiting Activity As Anticancer Agents Including Hedgehog-Dependent Cancer. J Med Chem. 2014; 57(15): 6531−6552. 10.1021/jm500561a 25025991 PMC4154712

[18] Guan Q, Feng D, Bai Z, Cui Y, Zuo D, Zhai M, et al Microwave-assisted synthesis, molecular docking and antiproliferative activity of (3/5-aryl-1,2,4-oxadiazole-5/3-yl)(3,4,5-trimethoxyphenyl)methanone oxime derivatives. Med Chem Comm. 2015; 6(8): 1484–1493.

[19] Kim KS, Kimball SD, Misra RN, Rawlins DB, Hunt JT, Xiao HY, et al Discovery of Aminothiazole Inhibitors of Cyclin-Dependent Kinase 2: Synthesis, X-ray Crystallographic Analysis, and Biological Activities. J. Med. Chem. 2002; 45(18): 3905–3927. 12190313 10.1021/jm0201520

[20] Wang XF, Ohkoshi E, Wang SB, Hamel E, Bastow KF, Morris-Natschke SL, et al Synthesis and biological evaluation of N-alkyl-N-(4-methoxyphenyl)pyridin-2-amines as a new class of tubulin polymerization inhibitors. Bioorg. Med. Chem. 2013; 21(3): 632–642. 10.1016/j.bmc.2012.11.047 23274123 PMC3546147

[21] Guan Q, Yang F, Guo D, Xu J, Jiang M, Liu C, et al Synthesis and biological evaluation of novel 3,4-diaryl-1,2,5-selenadiazol analogues of combretastatin A-4. Eur J Med Chem. 2014; 87: 1–9. 10.1016/j.ejmech.2014.09.046 25233100

[22] Xu Q, Qi H, Sun M, Zuo D, Jiang X, Wen Z; et al Synthesis and Biological Evaluation of 3-Alkyl-1,5-Diaryl-1H-Pyrazoles as Rigid Analogues of Combretastatin A-4 with Potent Antiproliferative Activity. PLoS One. 2015; 10(6): e0128710 10.1371/journal.pone.0128710 26061410 PMC4462585

[23] Das D, Sikdar P, Bairagi M. Recent developments of 2-aminothiazoles in medicinal chemistry. Eur J Med Chem. 2016; 109: 89–98. 10.1016/j.ejmech.2015.12.022 26771245

[24] Taguchi M, Kondo H, Inoue Y, Kawahata Y, Jinbo Y, Sakamoto F, et al Synthesis and antibacterial activity of new tetracyclic quinolone antibacterials. J Med Chem. 1992; 35(1): 94–99. 1310116 10.1021/jm00079a011

[25] Jiang Z, Chen Y, Yang C, Cao Y, Tao Y, Qin J, et al A fully diarylmethylene-bridged triphenylamine derivative as novel host for highly efficient green phosphorescent OLEDs. Org Lett. 2009; 11(7): 1503–1506. 10.1021/ol9001152 19256517

[26] Kabalka GW, Mereddy AR. Microwave promoted synthesis of functionalized 2-aminothiazoles. Tetrahedron Letters. 2006; 47: 5171–5172.

[27] Sato T, Tomaru K, Koide T, Masuda M, Yamamoto M, Miyazawa N; et al Synchronous lung and gastric cancers successfully treated with carboplatin and pemetrexed: a case report. J Med Case Rep. 2012; 6: 266 10.1186/1752-1947-6-266 22938085 PMC3441851

[28] Rasheed S, Nelson-Rees WA, Toth EM, Arnstein P, Gardner MB. Characterization of a newly derived human sarcoma cell line (HT-1080). Cancer. 1974; 33(4): 1027–1033. 4132053 10.1002/1097-0142(197404)33:4<1027::aid-cncr2820330419>3.0.co;2-z

