# Supplementary material for: Synthesis and bioevaluation of N,4-diaryl-1,3-thiazole-2-amines as tubulin inhibitors with potent antiproliferative activity
Source: PLoS One. 2017 Mar 23;12(3):e0174006. doi: 10.1371/journal.pone.0174006 (PMC5363846; doi:10.1371/journal.pone.0174006)

Synthesis and bioevaluation of *N*,4-diaryl-1,3-thiazole-2-amines as tubulin inhibitors with potent antiproliferative activity

Maolin Sun[1¶], Qile Xu[1¶], Jingwen Xu[2], Yue Wu[1], Yueting Wang[1], Daiying Zuo[2], Qi Guan[1], Kai Bao[1,3], Jian Wang[1*], Yingliang Wu[2], Weige Zhang[1]*

[1] Key Laboratory of Structure-Based Drug Design and Discovery, Ministry of Education, Shenyang Pharmaceutical University, Shenyang, China.

[2] Department of Pharmacology, Shenyang Pharmaceutical University, Shenyang, China.

[3] Gordon Center for Medical Imaging, Division of Nuclear Medicine and Molecular Imaging, Department of Radiology, Massachusetts General Hospital and Harvard Medical School, Boston, USA.

* Corresponding authors:

E-mail addresses: zhangweige2000@sina.com (W. Zhang)

jianwang@syphu.edu.cn (J. Wang)

¶ These authors contributed equally to this work.

List of content

(1). Synthesis.

1. General synthetic procedures for aryl thioamides 14.

2. General synthetic procedures for α-bromoacetophenones 16.

(2). Contents: ^{1}H-NMR and ^{13}C-NMR spectra of all target compounds.

(1). Synthesis

1. General synthetic procedures for aryl thioamides 14.

A mixture of aniline derivatives **11** (1.5 mmol), carbon disulfide (1.8 mmol), and triethylamine (4.5 mmol) was stirred at room temperature. After the reaction was completed, the precipitated crystals were collected and washed with ether to yield **12** as a slightly pale yellow or yellow crystals. To a stirred and ice cooled suspension of dithiocarbamate (2 mmol) in ethylacetate, was added triethylamine (3 mmol). To this was then added iodine (2 mmol) pinch wise over a period of 10-15 minutes to yield phenylisothiocyanate. During this period precipitation of elemental sulfur and triethylammonium iodide salt was observed. After complete addition of iodine, 25% aqueous NH_3 (2.5 mL) was added drop wise to the stirred reaction mixture to give 1-phenylthiourea. After the reaction was completed, the precipitate was filtered and washed with EtOAc, the solvent was then evaporated under reduced pressure. The residue was used for the next step without further purification.

2. General synthetic procedures for α-bromoacetophenones 16.

A solution of appropriately substituted acetophenone (10 mmol) in 80 mL of $CHCl_3$ was added in one portion to a vigorously stirred, refluxing suspension of 21mmol of $CuBr_2$ in 65 mL of ethyl acetate. The reaction was practically complete after refluxing for 1.5 h, as indicated by the conversion of $CuBr_2$ (black) into CuBr (white), lack of HBr evolution, and TLC (ethyl acetate : cyclohexane = 1:4). The solids were removed by filtration, washing with ethyl acetate. The residue from evaporation was distributed between ethyl acetate (2 × 350 mL) and semisaturated $NaHCO_3$ (2×200 mL). The organic layers were washed with semisaturated brine (200 mL), dried (Na_2SO_4), and evaporated in vacuo. The crude residue was passed through a quick column, eluting with ethyl acetate-cyclohexane (20:80, v/v) and used as such for the next step.

(2). Contents: ^{1}H-NMR and ^{13}C-NMR spectra of all target compounds.

N-(3,4,5-trimethoxyphenyl)-4-(4-methyphenyl)-1,3-thiazol-2-amine (**10a**).

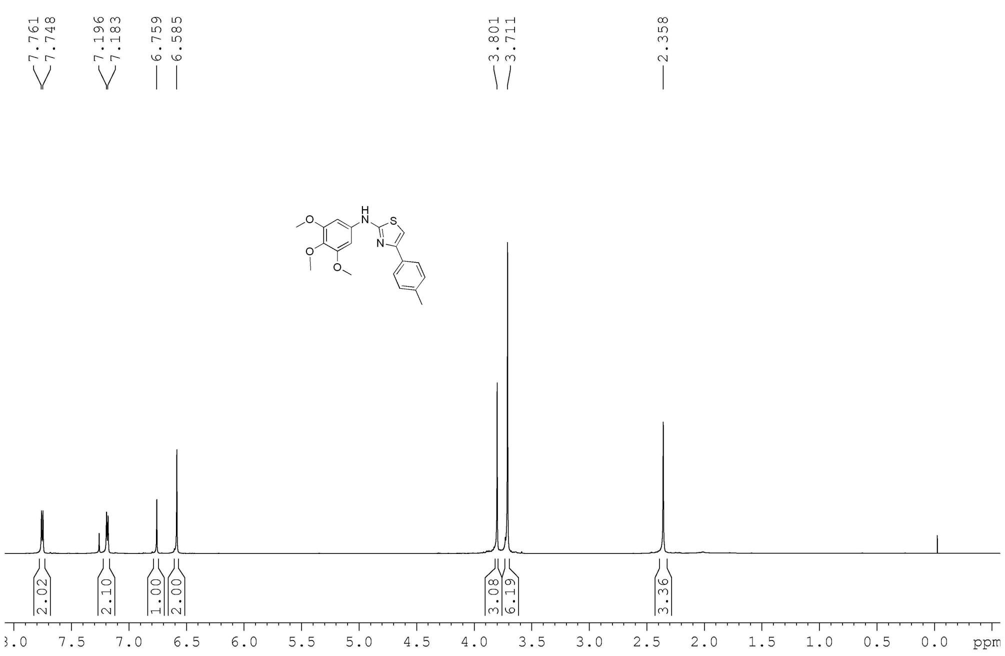

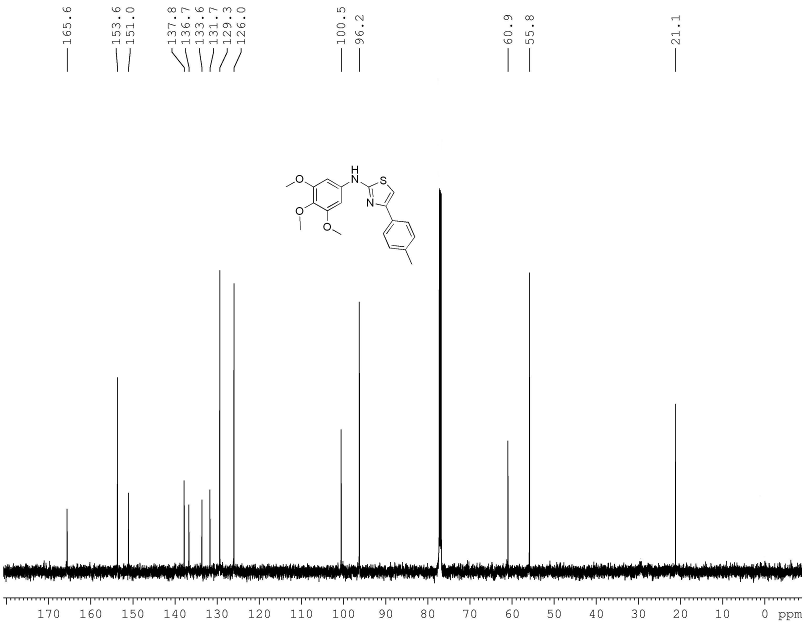

N-(3,4,5-trimethoxyphenyl)-4-(4-methoxyphenyl)-1,3-thiazol-2-amine (**10b**).

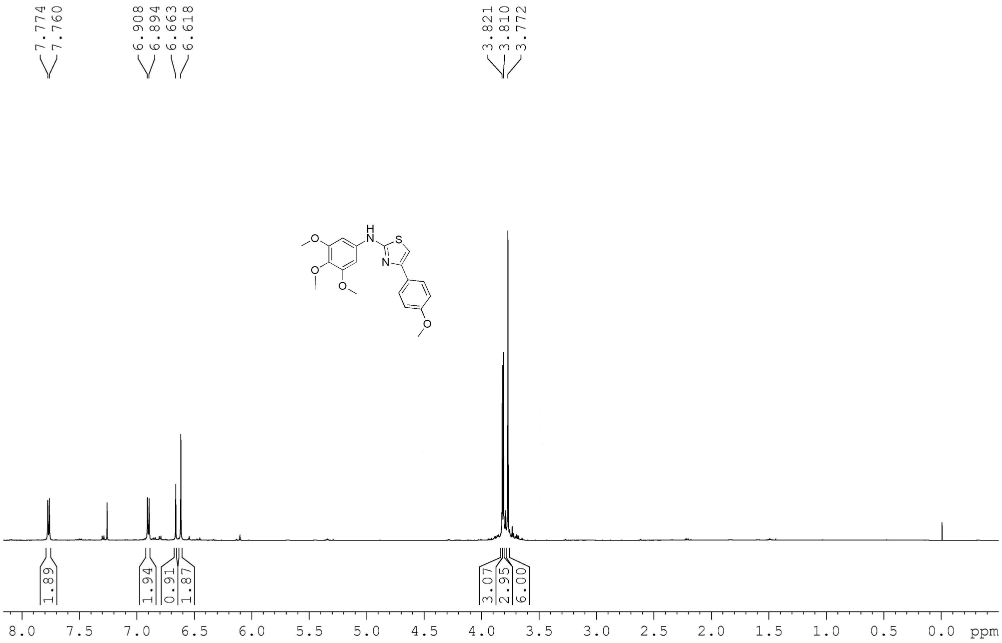

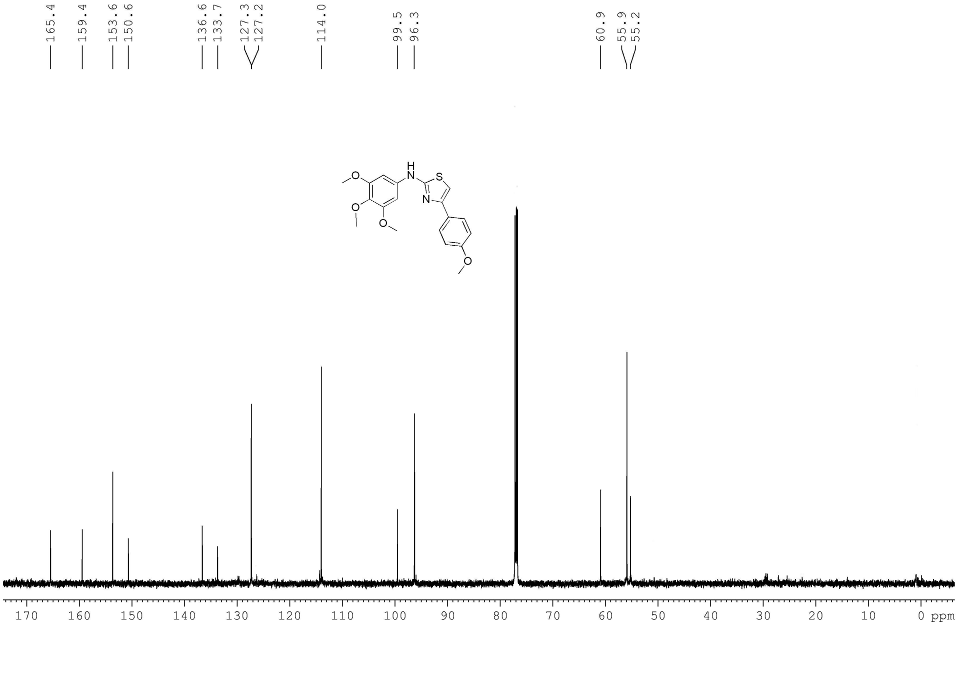

N-(3,4,5-trimethoxyphenyl)-4-(4-fluorophenyl)-1,3-thiazol-2-amine (**10c**).

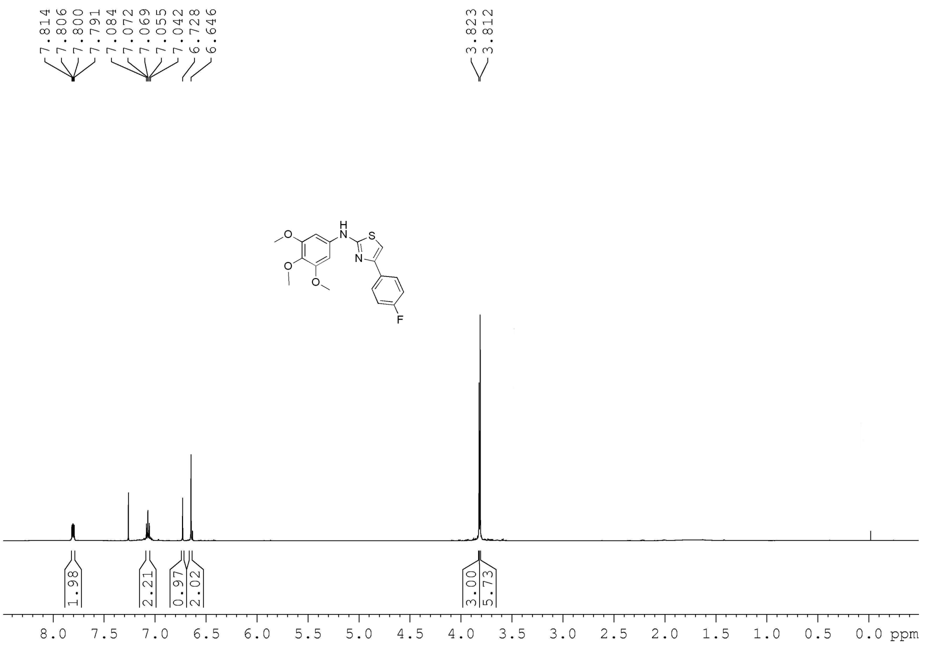

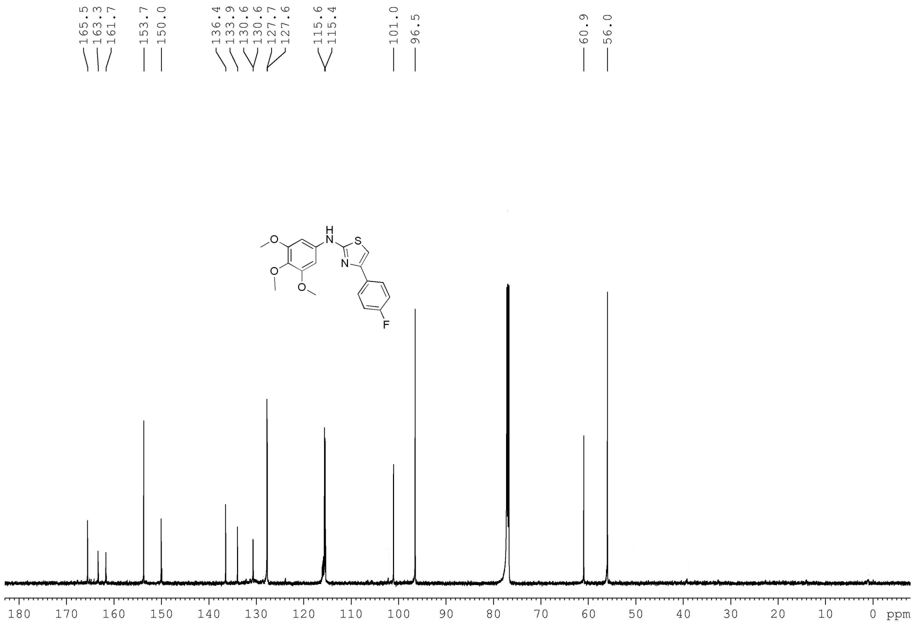

N-(3,4,5-trimethoxyphenyl)-4-(4-chlorophenyl)-1,3-thiazol-2-amine (**10d**).

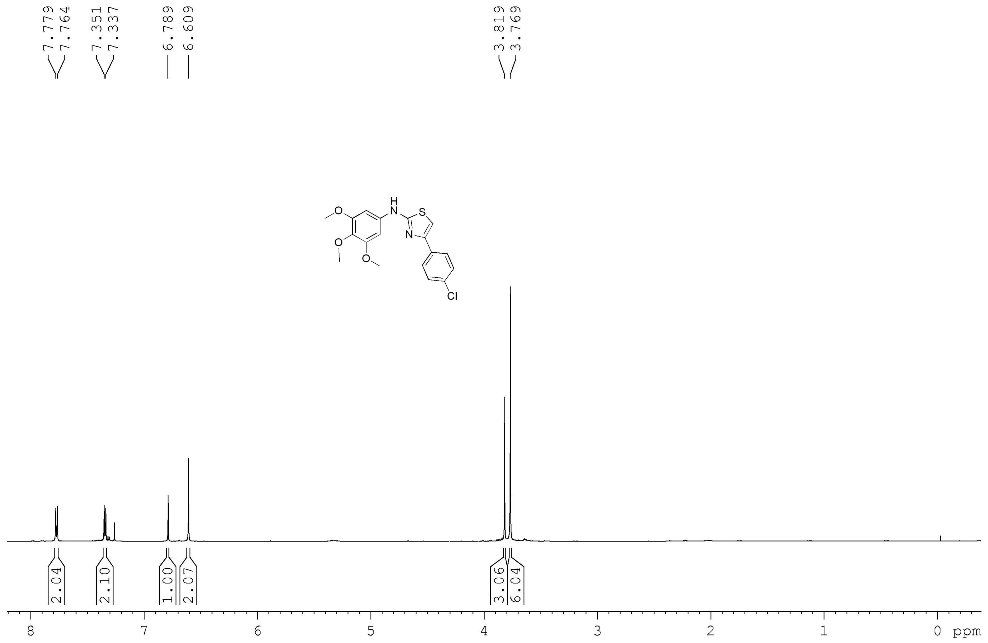

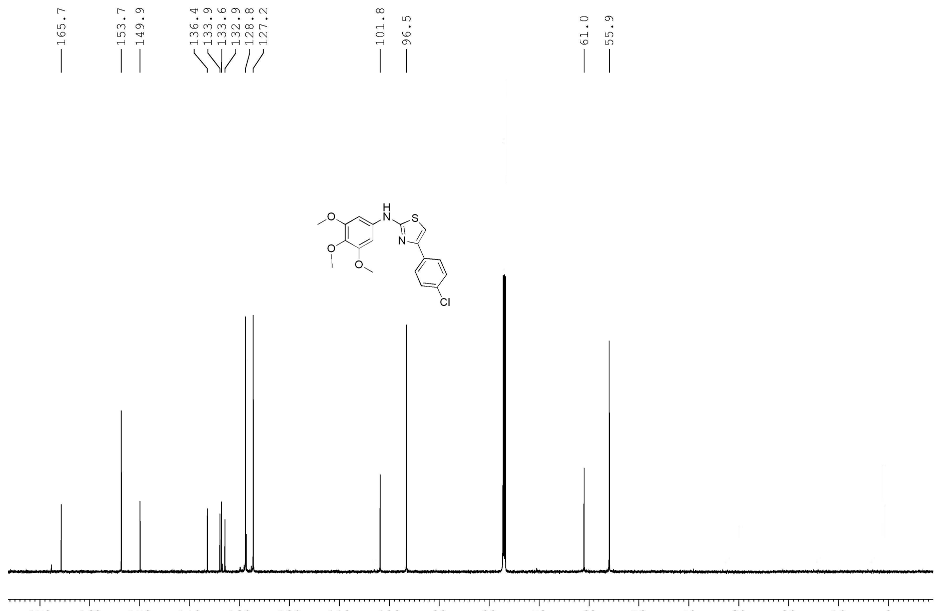

N-(3,4,5-trimethoxyphenyl)-4-(4-bromophenyl)-1,3-thiazol-2-amine (**10e**).

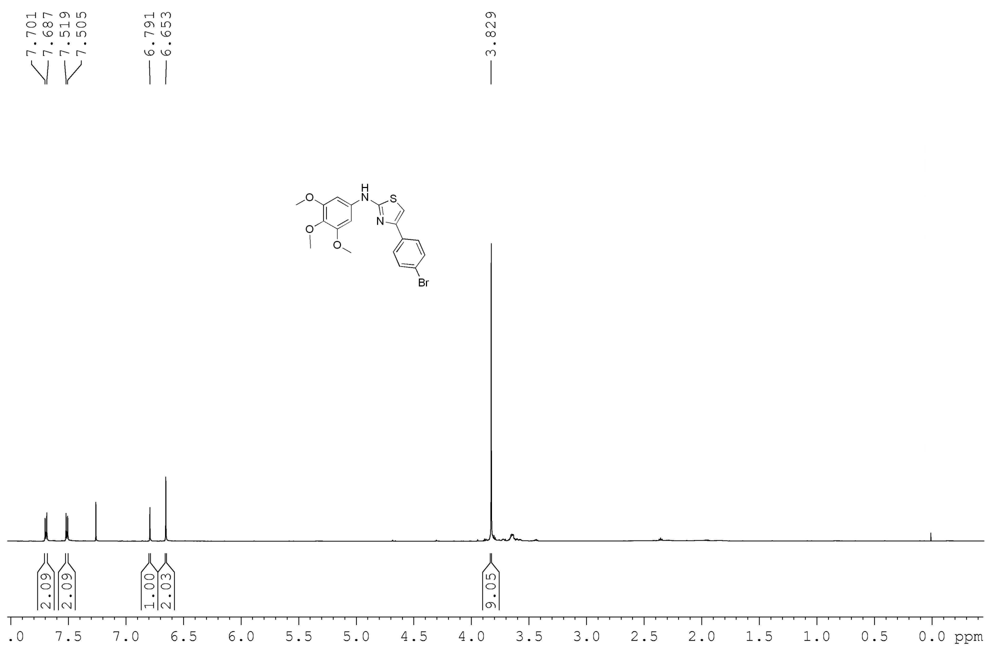

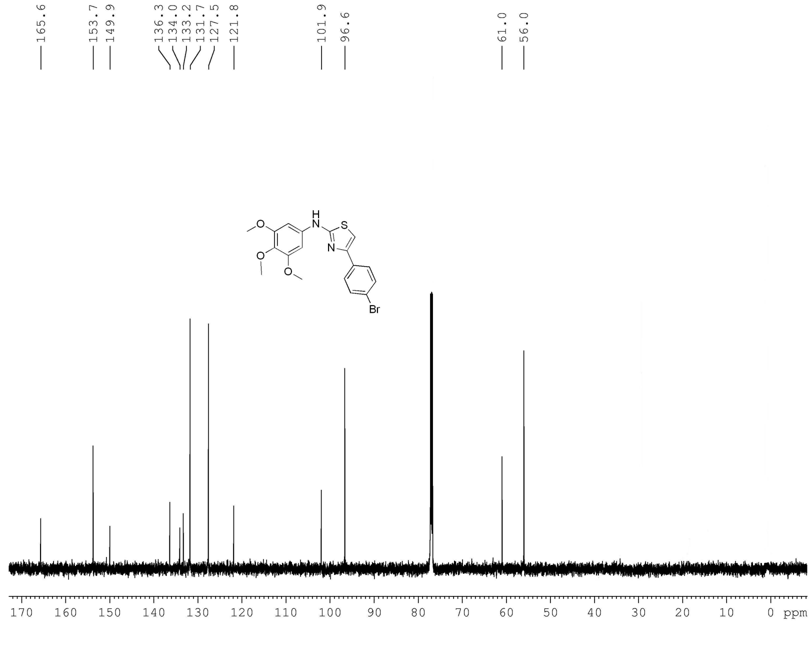

N-(3,4,5-trimethoxyphenyl)-4-(4-nitrophenyl)-1,3-thiazol-2-amine (**10f**).

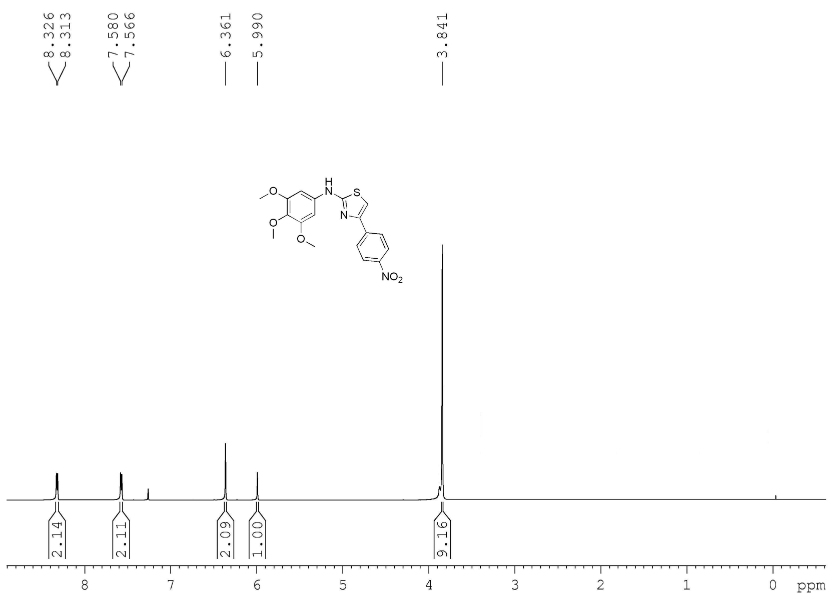

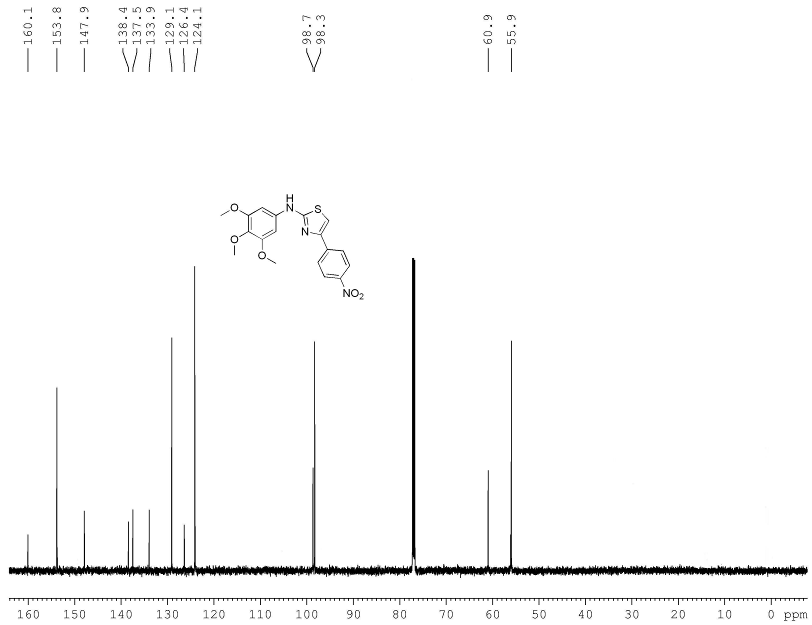

N-(3,4,5-trimethoxyphenyl)-4-(3,4-dimethoxyphenyl)-1,3-thiazol-2-amine (**10g**).

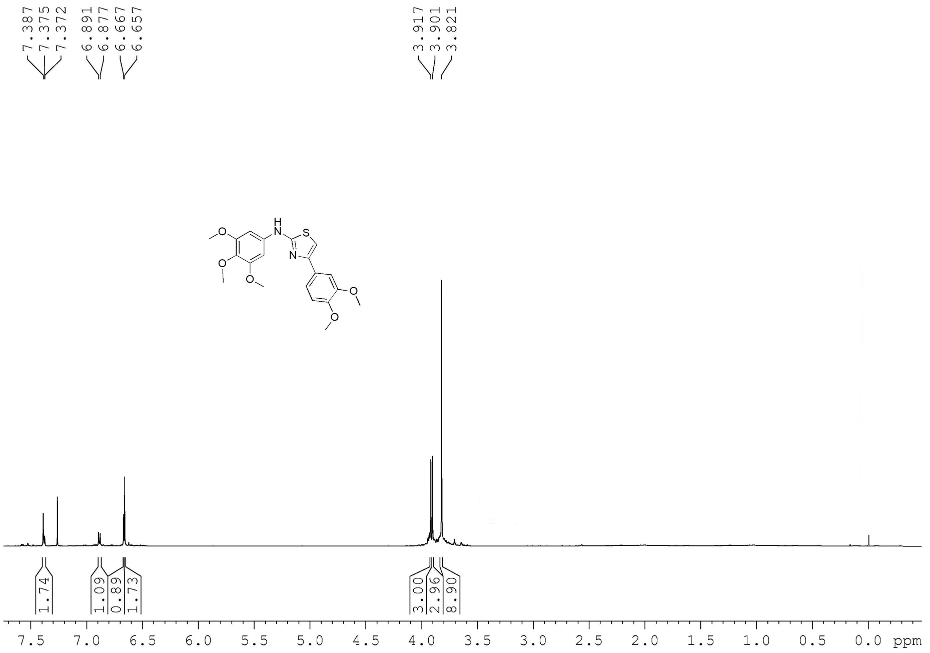

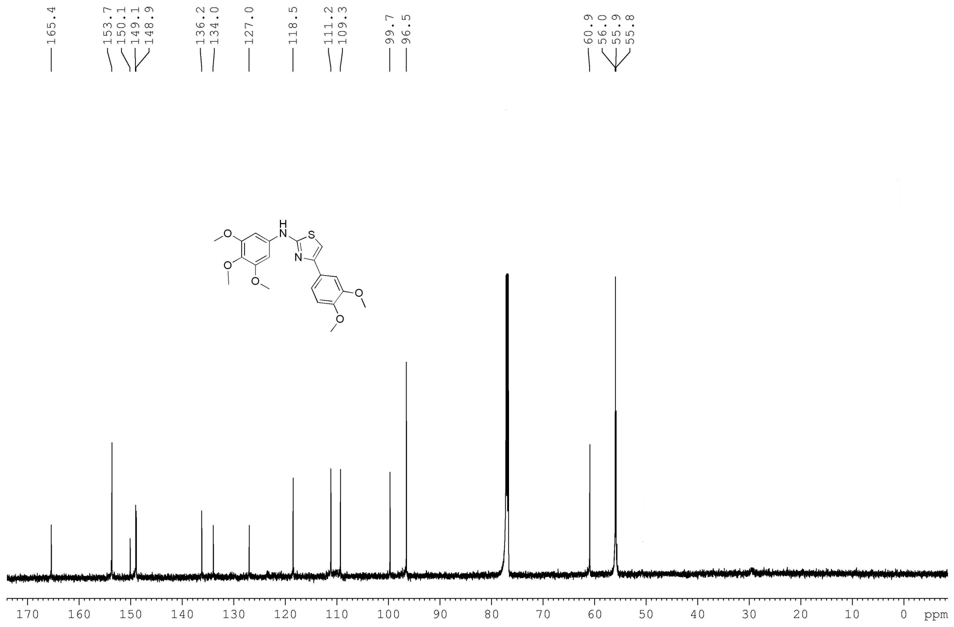

N-(3,4,5-trimethoxyphenyl)-4-(3-fluoro-4-methoxyphenyl)-1,3-thiazol-2-amine (**10h**).

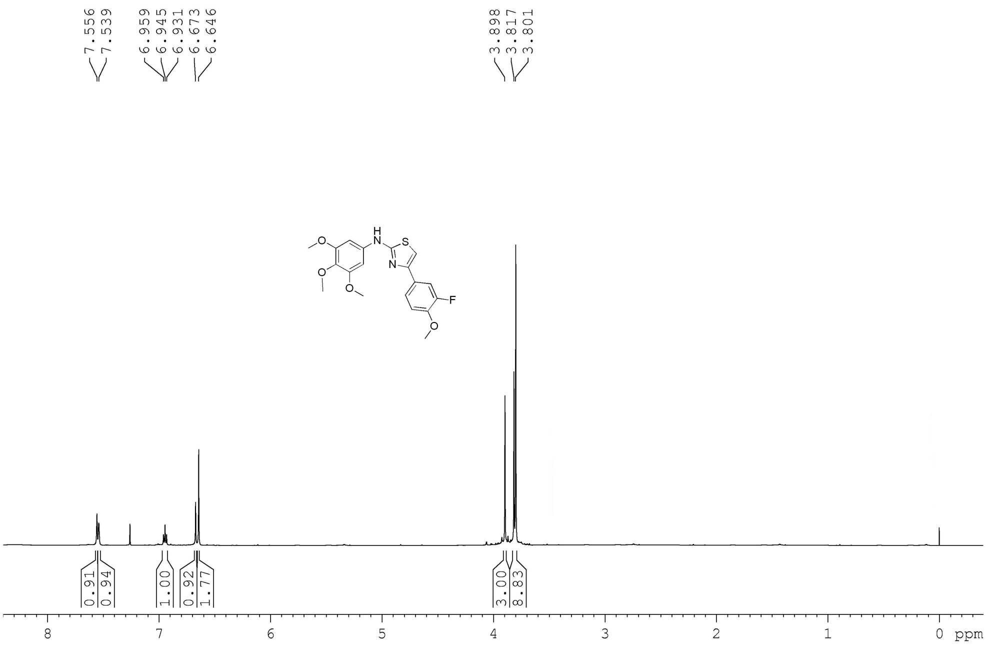

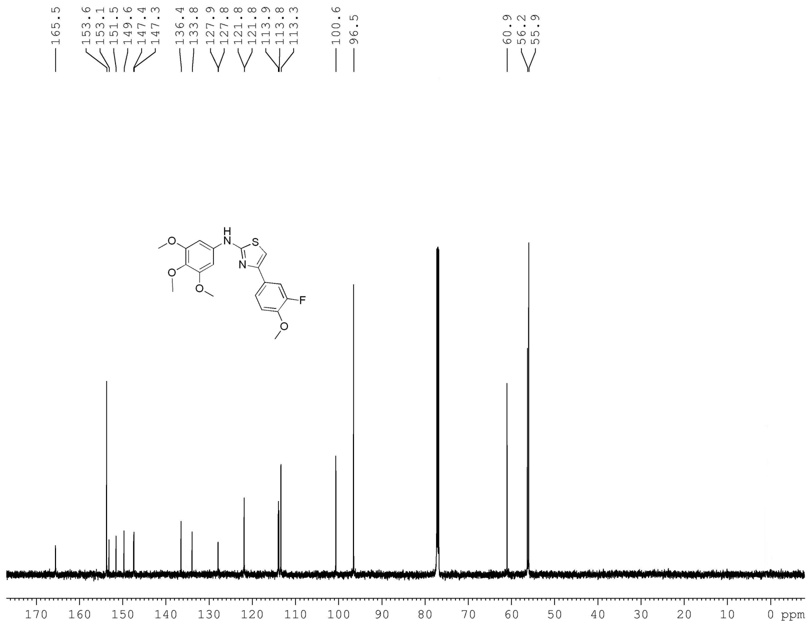

N-(3,4,5-trimethoxyphenyl)-4-(3-nitro-4-methoxyphenyl)-1,3-thiazol-2-amine (**10i**).

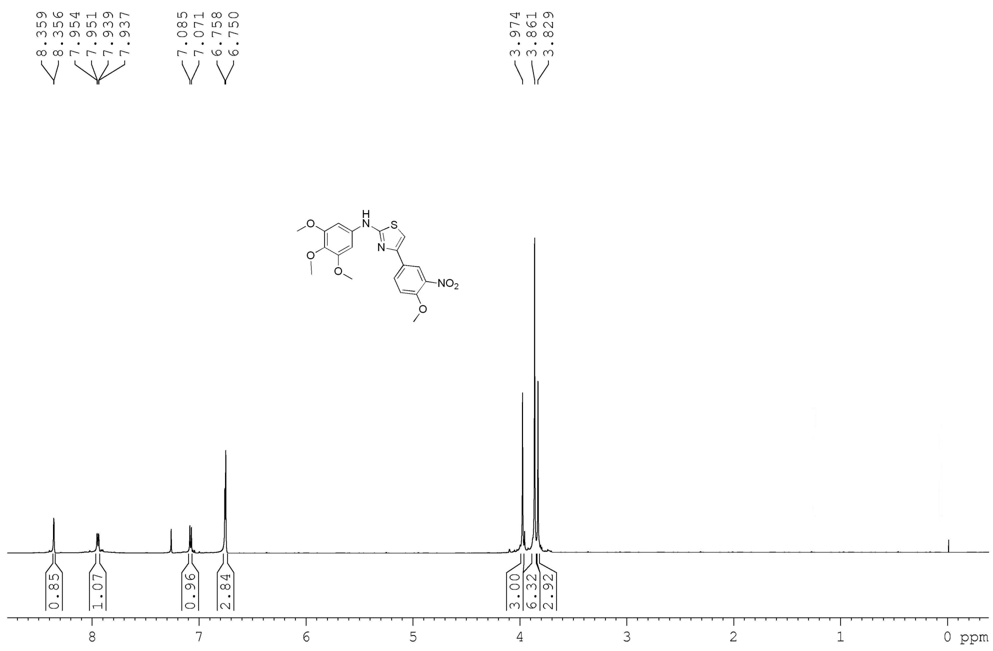

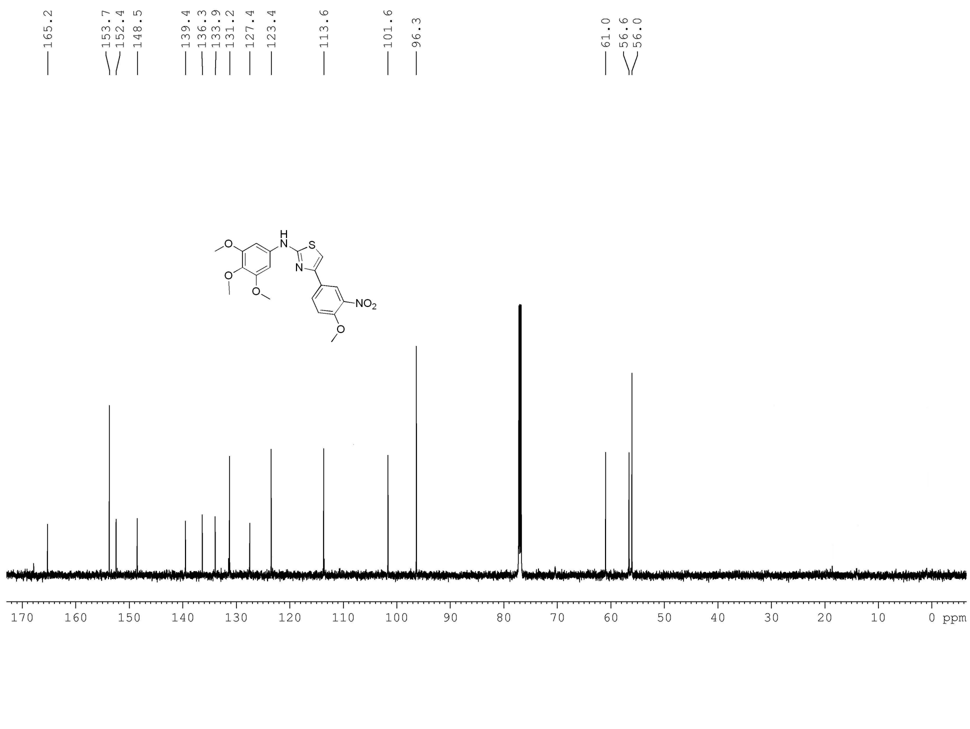

N-(3,4,5-trimethoxyphenyl)-4-(3-amino-4-methoxyphenyl)-1,3-thiazol-2-amine (**10j**).

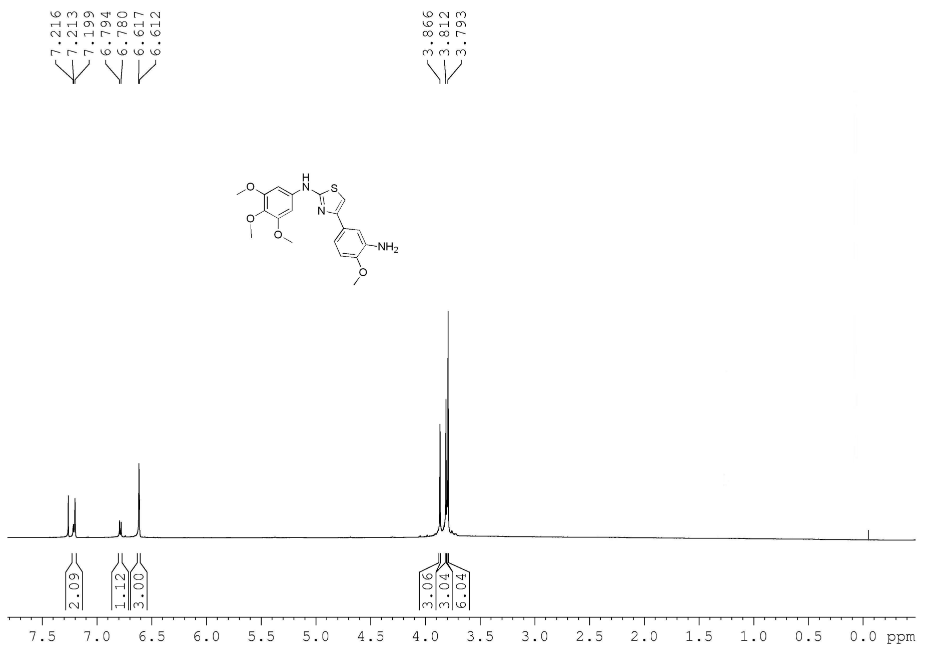

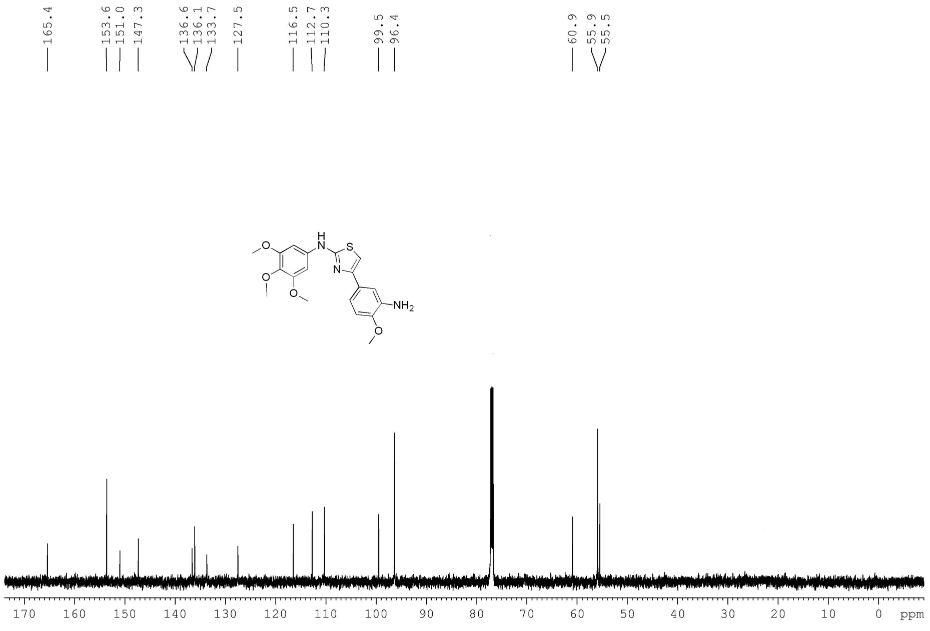

N-(3,4,5-trimethoxyphenyl)-4-(3-benzyloxy-4-methoxyphenyl)-1,3-thiazol-2-amine (**10k**).

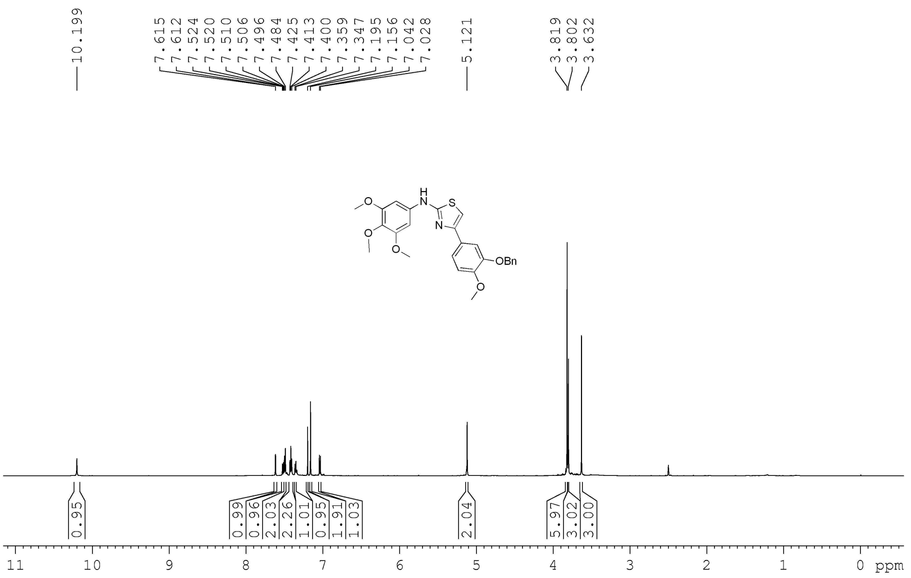

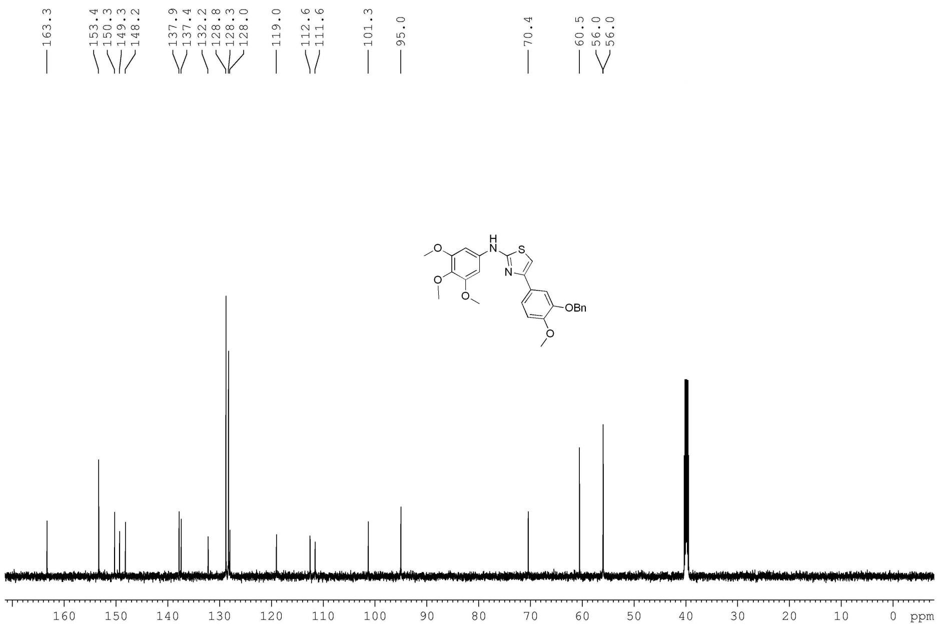

N-(3,4,5-trimethoxyphenyl)-4-(3-hydroxy-4-methoxyphenyl)-1,3-thiazol-2-amine (**10l**).

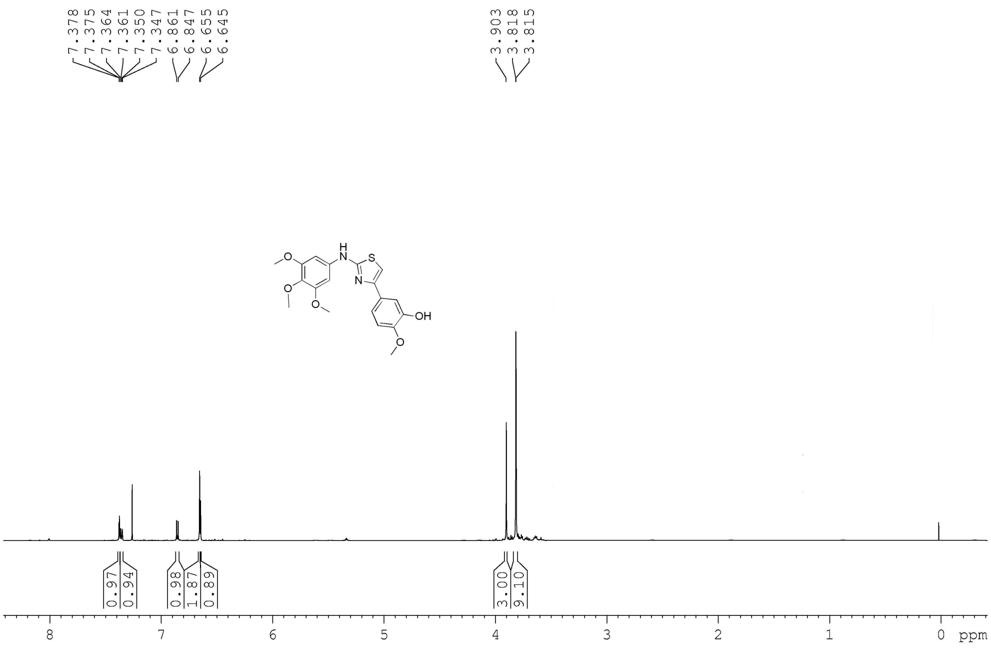

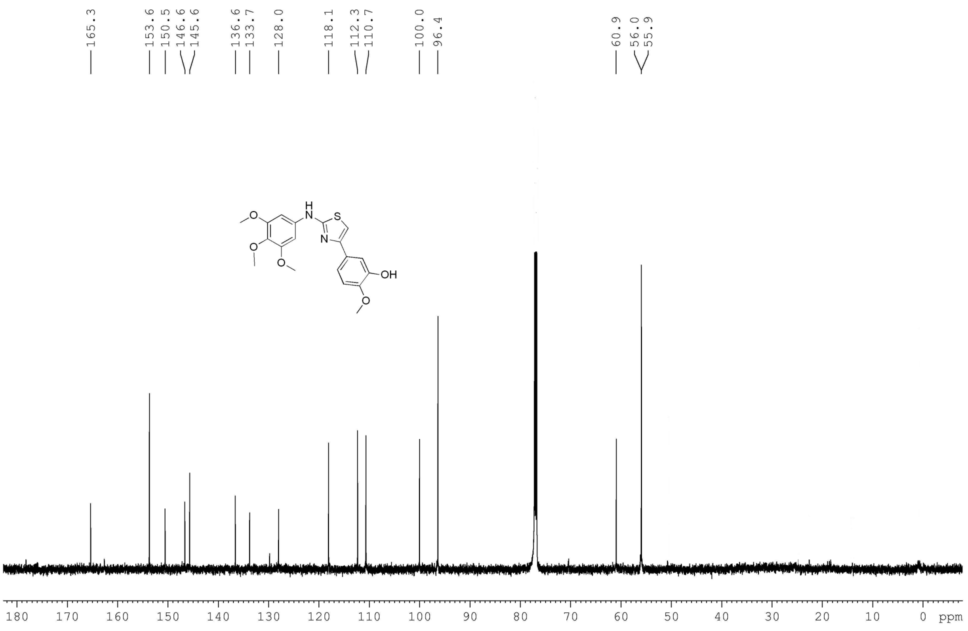

N-(3,4,5-trimethoxyphenyl)-4-(3,4-difluorophenyl)-1,3-thiazol-2-amine (**10m**).

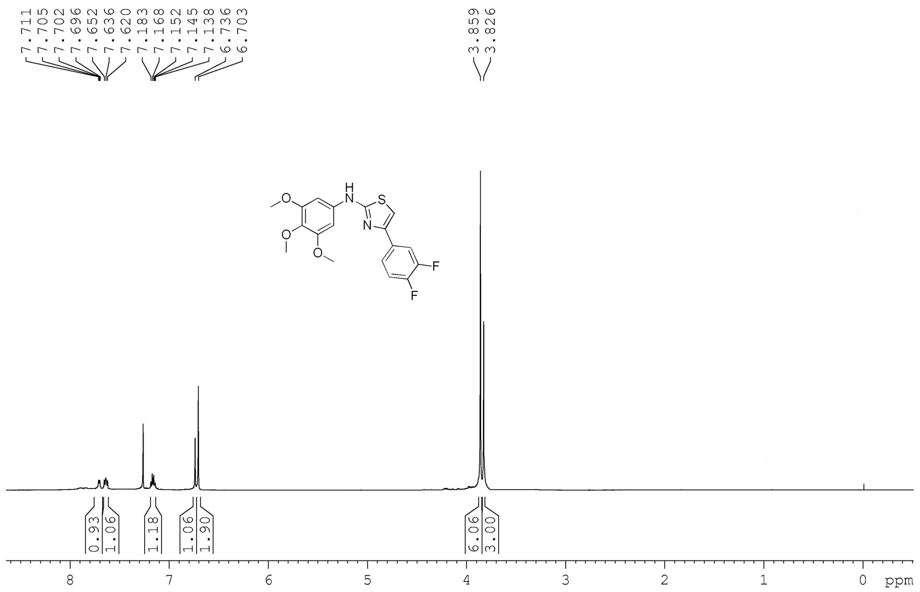

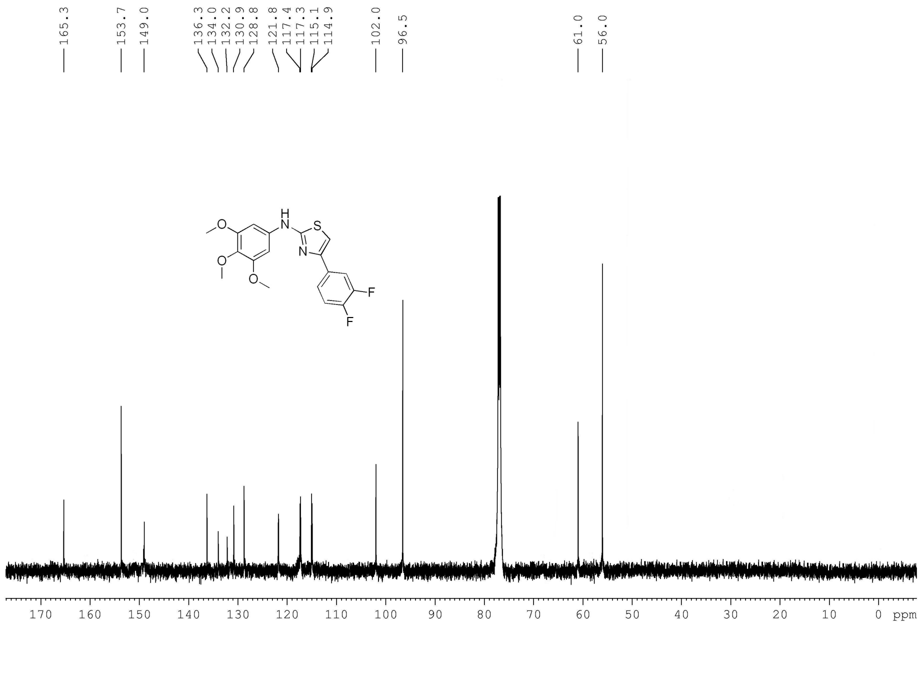

N-(3,5-dimethoxyphenyl)-4-(4-methoxyphenyl)-1,3-thiazol-2-amine (**10n**).

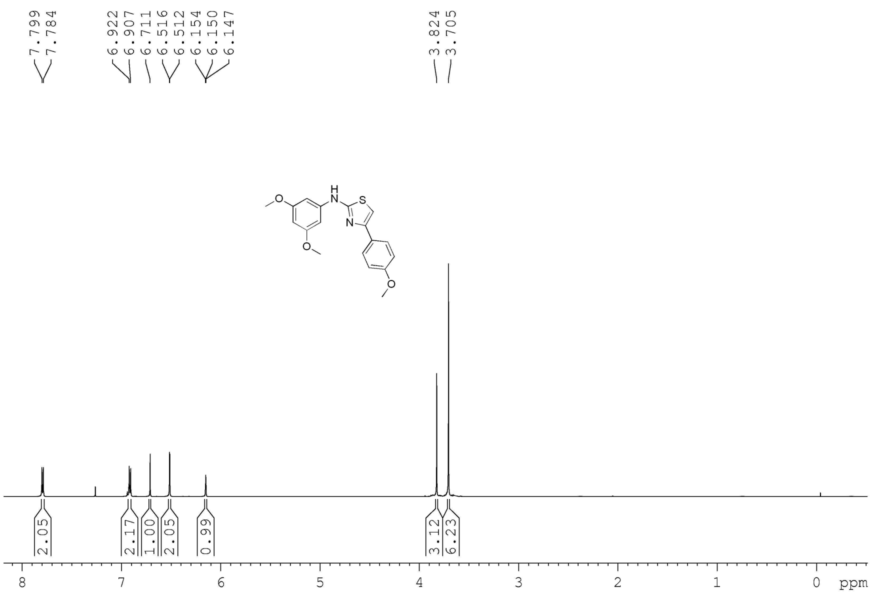

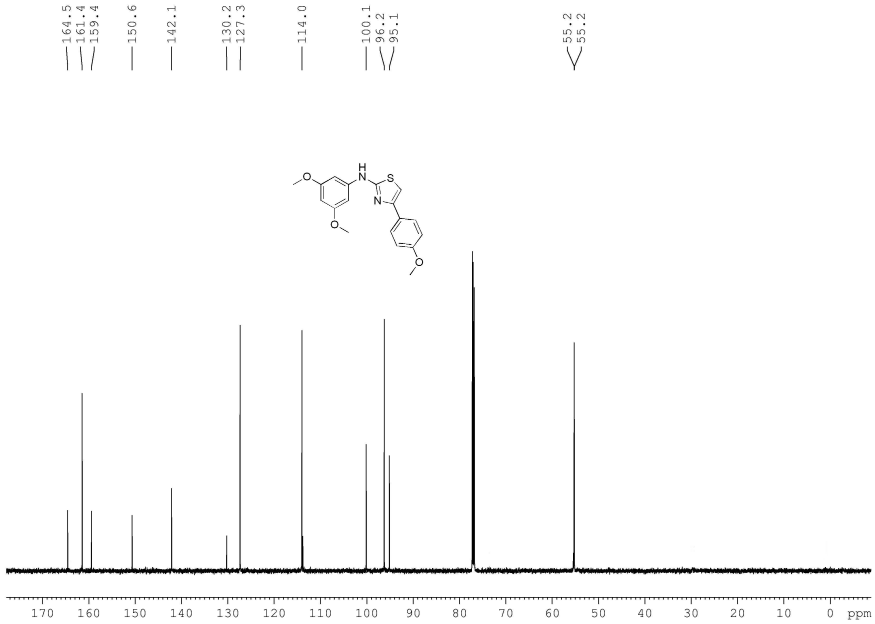

N-(4-methoxyphenyl)-4-(3,4,5-trimethoxyphenyl)-1,3-thiazol-2-amine (**10o**).

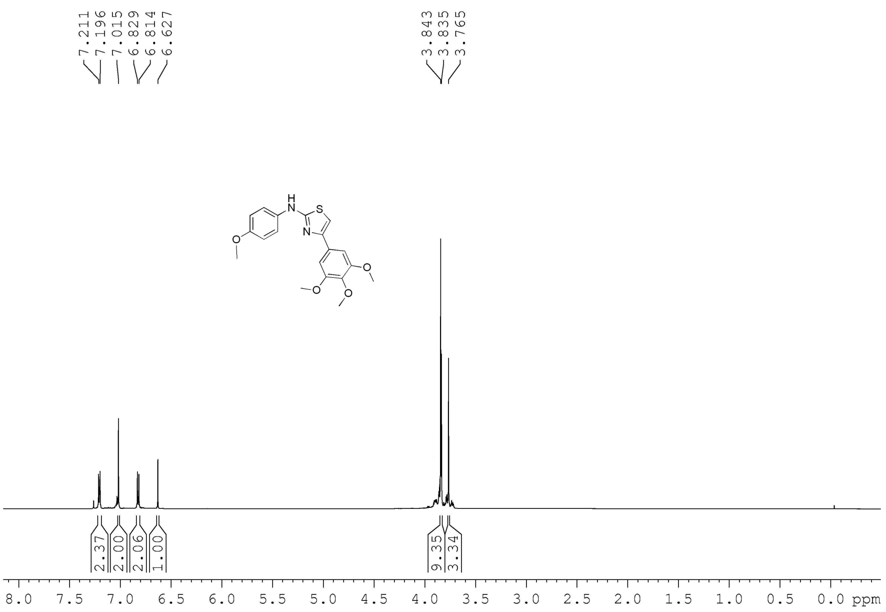

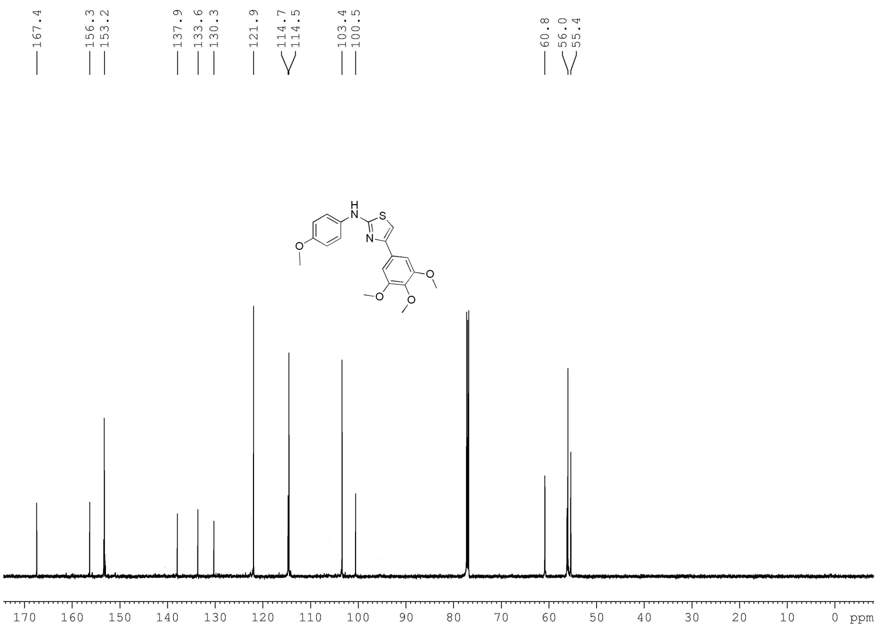

N-(3,4-dimethphenyl)-4-(3-nitro-4-methoxyphenyl)-1,3-thiazol-2-amine (**10p**).

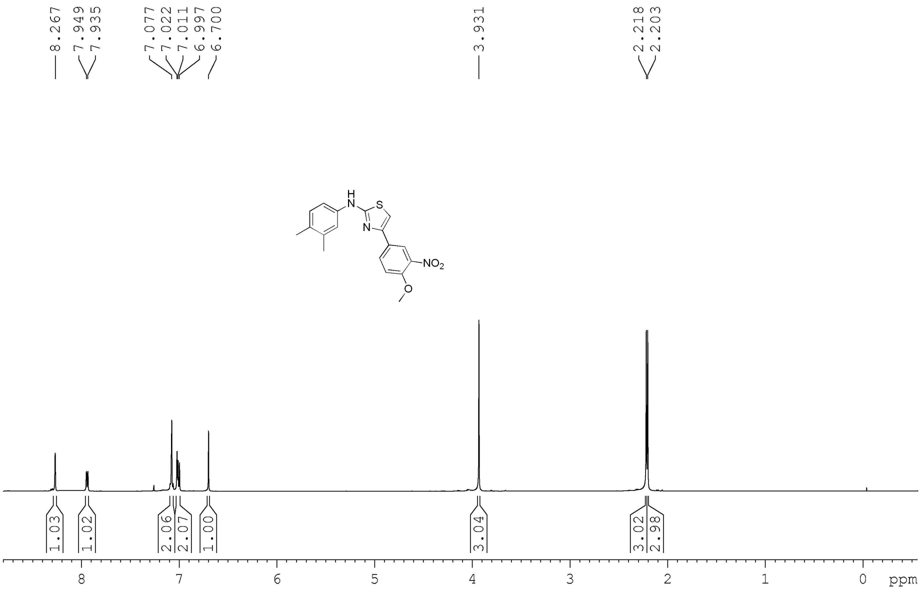

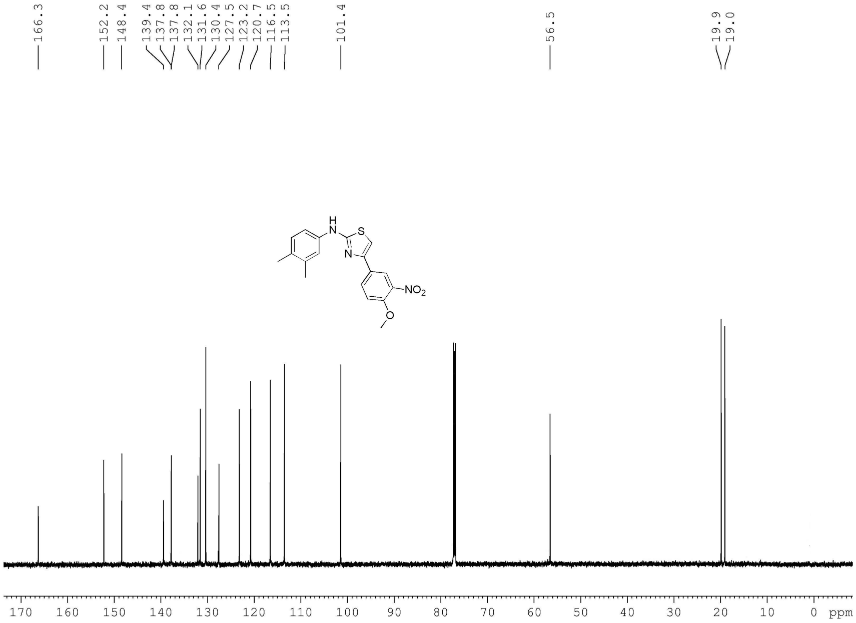

N-(3,4-dimethphenyl)-4-(3-amino-4-methoxyphenyl)-1,3-thiazol-2-amine (**10q**).

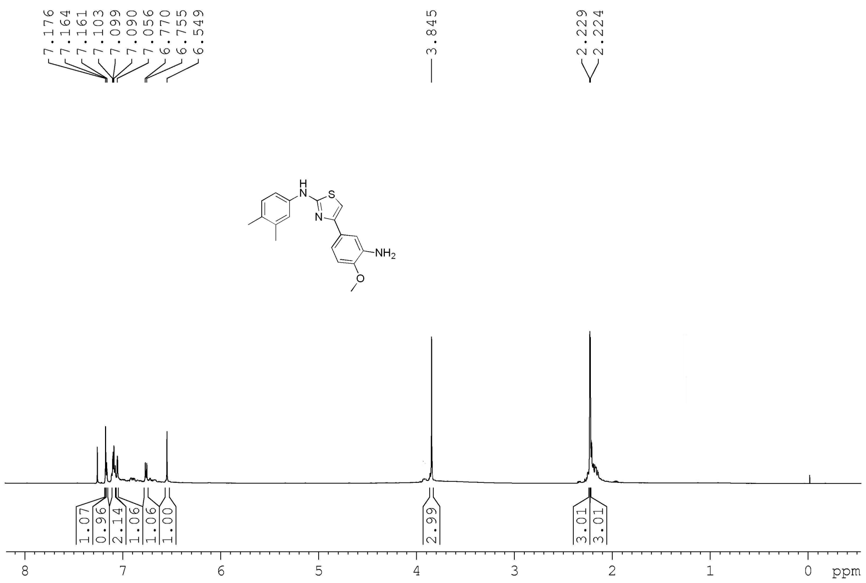

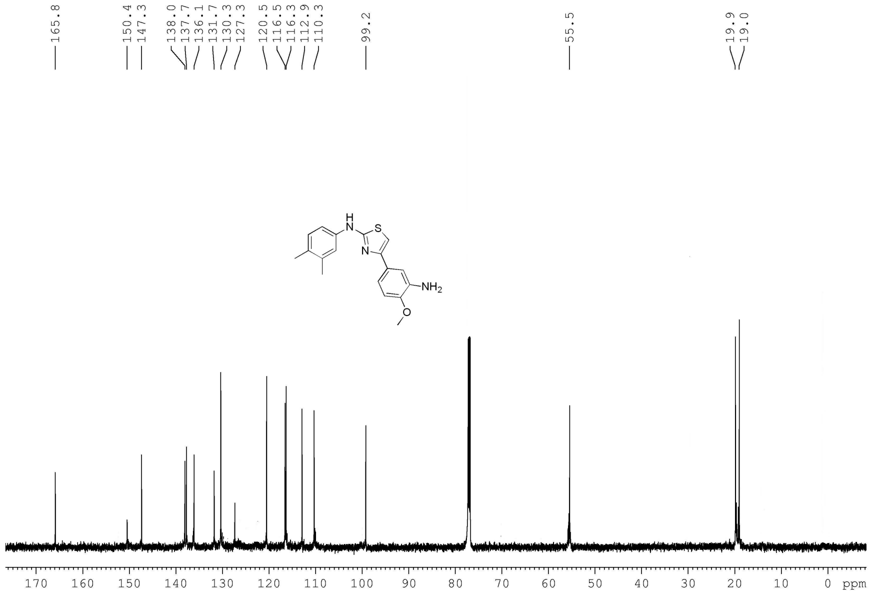

N-(2,4-dimethoxyphenyl)-4-(4-methphenyl)-1,3-thiazol-2-amine (**10r**).

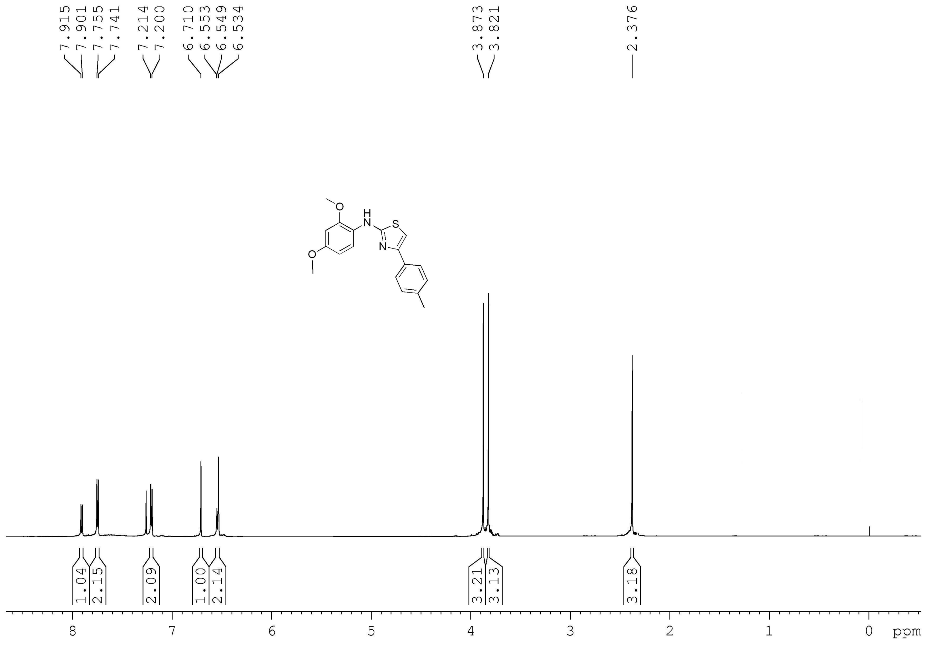

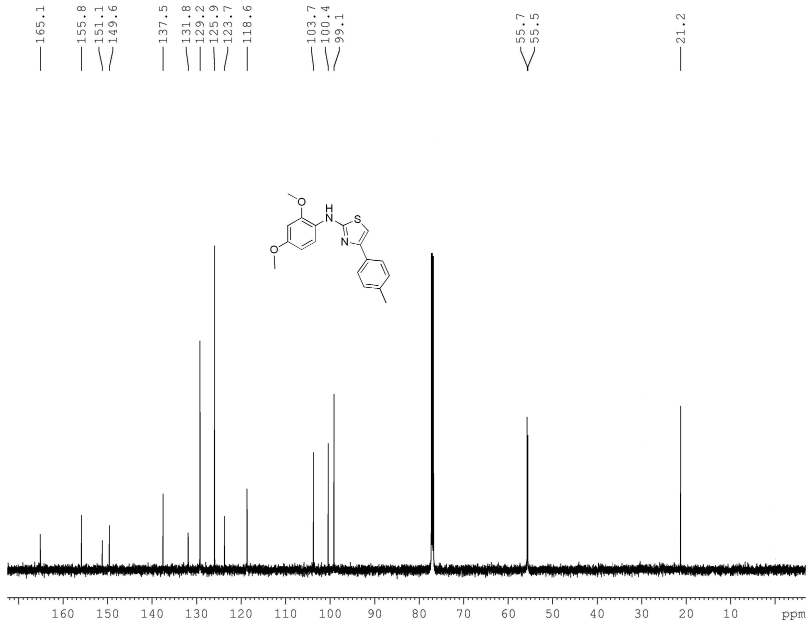

N-(2,4-dimethoxyphenyl)-4-(4-methoxyphenyl)-1,3-thiazol-2-amine (**10s**).

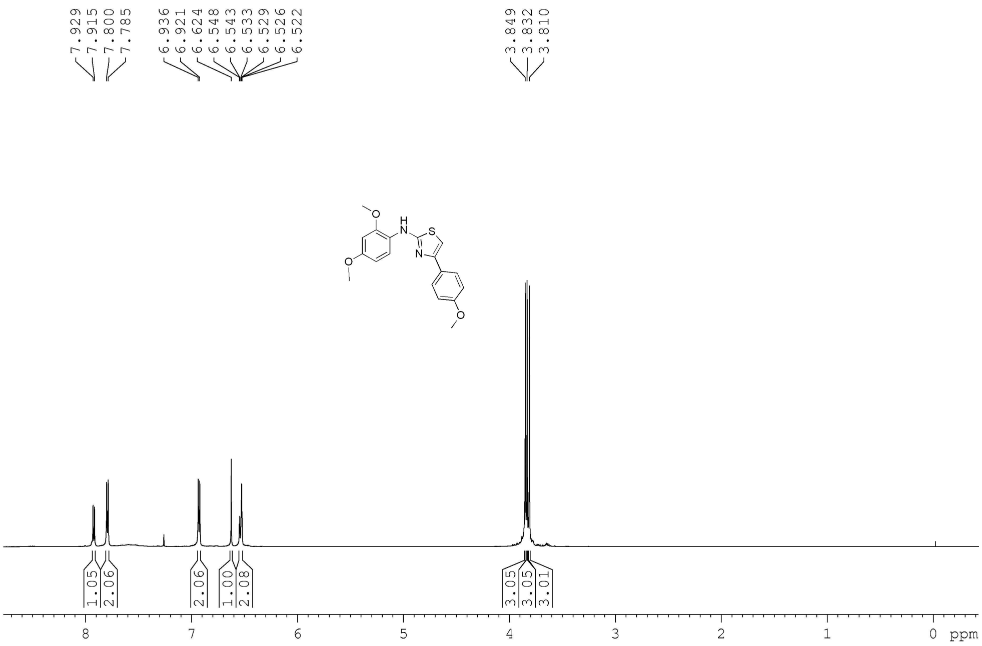

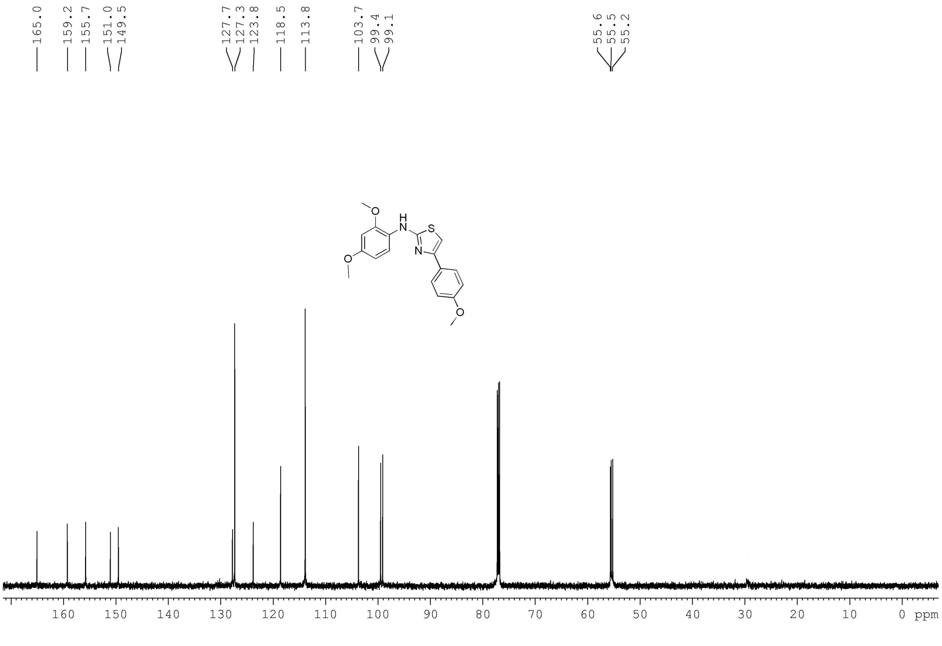

N-(2,4-dimethoxyphenyl)-4-(2,4-dimethoxyphenyl)-1,3-thiazol-2-amine (**10t**).

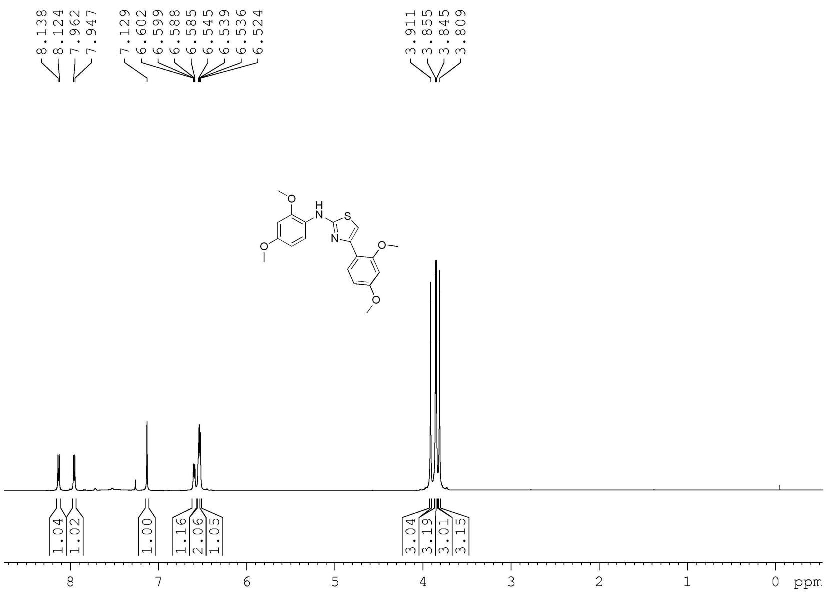

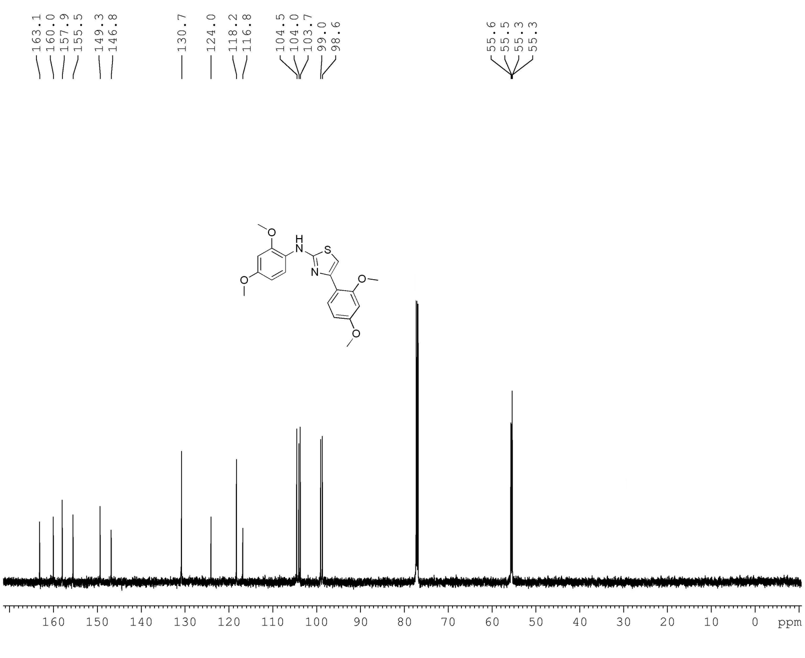

N-(2,4-dimethoxyphenyl)-4-(4-methoxyphenyl)-*N*-methyl-1,3-thiazol-2-amine (**10u**).

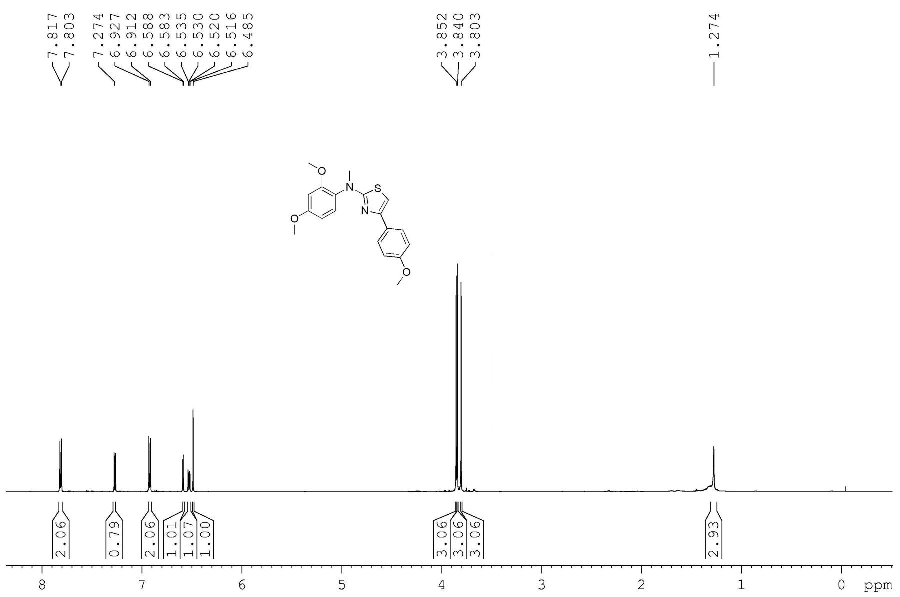

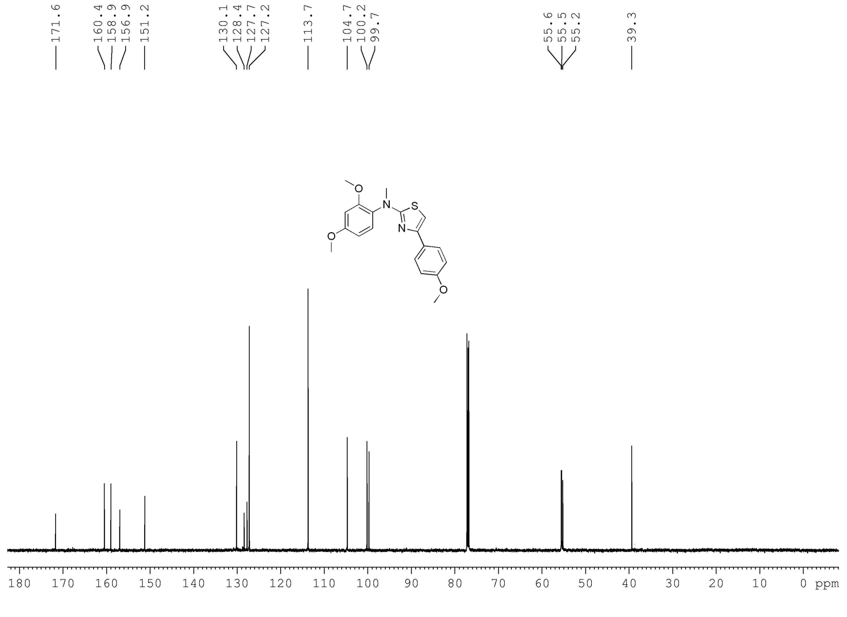

N-(2,4-dimethoxyphenyl)-4-(4-methoxyphenyl)-*N*-acetyl-1,3-thiazol-2-amine (**10v**).

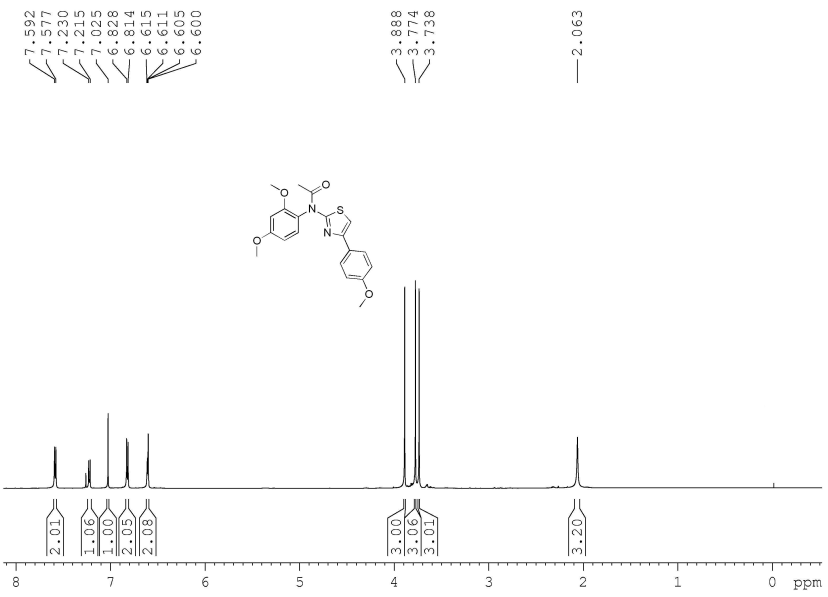

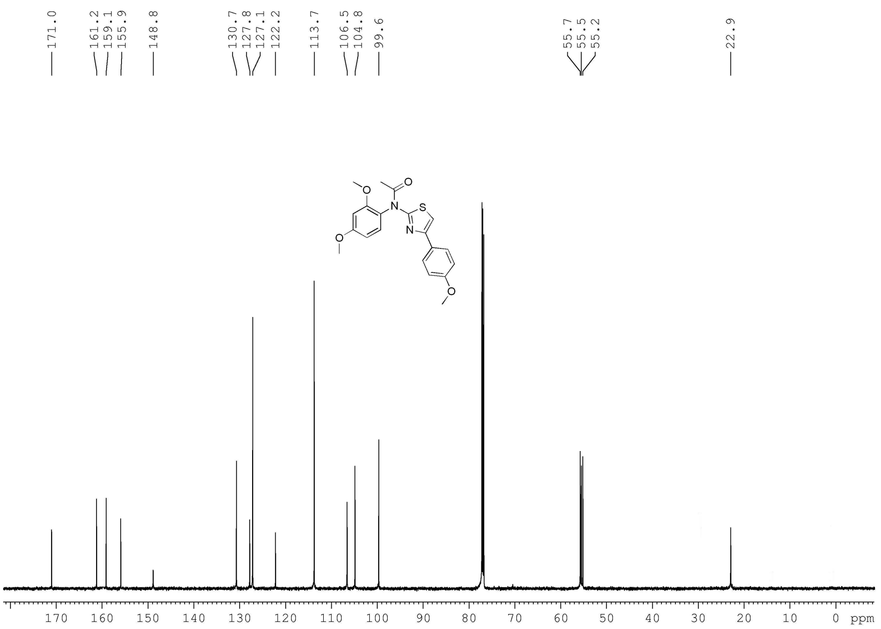

Supplement: S1 File — (1). Synthesis. 1. General synthetic procedures for aryl thioamides 14. 2. General synthetic procedures for α-bromoacetophenones 16. (2). Contents: 1H-NMR and 13C-NMR spectra of all target compounds. (PDF) [file pone.0174006.s001.pdf]
